# Changes in the Membrane-Associated Proteins of Exosomes Released from Human Macrophages after *Mycobacterium tuberculosis* Infection

**DOI:** 10.1038/srep37975

**Published:** 2016-11-29

**Authors:** Gustavo Diaz, Lisa M. Wolfe, Nicole A. Kruh-Garcia, Karen M. Dobos

**Affiliations:** 1 Department of Microbiology, Immunology and Pathology, Colorado State University, Fort Collins, Colorado, United States of America; 2 Proteomics and Metabolomics Facility, Colorado State University, Fort Collins, Colorado, United States of America

## Abstract

Tuberculosis (TB) is the deadliest infectious disease worldwide. One obstacle hindering the elimination of TB is our lack of understanding of host-pathogen interactions. Exosomes, naturally loaded with microbial molecules, are circulating markers of TB. Changes in the host protein composition of exosomes from *Mycobacterium tuberculosis* (*Mtb*)-infected cells have not been described, can contribute to our understanding of the disease process, and serve as a direct source of biomarkers or as capture targets to enrich for exosomes containing microbial molecules. Here, the protein composition of exosomes from *Mtb*-infected and uninfected THP-1-derived macrophages was evaluated by tandem-mass-spectrometry and differences in protein abundances were assessed. Our results show that infection with *Mtb* leads to significant changes in the protein composition of exosomes. Specifically, 41 proteins were significantly more abundant in exosomes from *Mtb-*infected cells; 63% of these were predicted to be membrane associated. Thus, we used a novel biotinylation strategy to verify protein localization, and confirmed the localization of some of these proteins in the exosomal membrane. Our findings reveal another important scenario where *Mtb* could be influencing changes in host cells that unveil new features of the host-pathogen interaction and may also be exploited as a source of biomarkers for TB.

Tuberculosis (TB) is the deadliest infectious disease worldwide; despite available treatment, 1.5 million people died from TB during 2014^1^. Additionally, during the last 5 years the global incidence of TB remained above 100 cases/100,000 population per year^2^. A major obstacle to control TB is our lack of understanding of host-pathogen interaction. Upon infection *Mycobacterium tuberculosis* (*Mtb,* the causal agent of TB) invades and grows mainly inside alveolar macrophages^3^. It is known that *Mtb* is able to modulate several mechanisms in the host to its favor, such as the blockage of phagosome maturation^4^, the disruption of the phagosome^5^ and the immune response^6^,^7^. The definitive explanation of these phenomena still needs to be defined. Exosomes are a type of extracellular vesicle involved in cell to cell communication^8^,^9^ and may unveil additional information about the mechanisms of interaction between *Mtb* and host cells.

Another scenario where exosomes are equally relevant is in biomarker discovery and diagnosis of TB. Historically, the identification of patients with *Mtb* infection is confirmed through the visualization of acid-fast bacilli in infected sputum samples using light microscopy. This test has shown very low sensitivity (50% to 60%), and is even lower in cases of HIV co-infected TB patients which account globally for the 51% of TB cases^1^. Of equal concern is the reservoir of latently infected individuals, comprising a third of the global population, from which 5% to 15% will develop active disease especially within the first 5 years of infection^10^. The available strategies to detect latent TB infection (LTBI) include tuberculin skin test (TST) or interferon gamma release assays (IGRA); both rely on sufficient T-cell based immune response against *Mtb* antigens^11^. In the case of TST, people that have received BCG-vaccination often have false positive results. This is important considering that roughly 90% of the countries have a policy of universal BCG vaccination^12^. IGRA is a suitable alternative designed to detect T-cell mediated gamma-interferon release in response to specific *Mtb-complex* antigens. Both tests show low sensitivity against HIV infected patients and neither can discriminate LTBI from TB or identify LTBI at high risk to develop TB^11^. As such, additional biomarker tests for LTBI identification and TB diagnosis are urgently needed.

Exosomes have emerged as a promising alternative source of biomarkers for several diseases, since they can be obtained from almost all biological fluids^8^,^9^,^13^. Exosome composition depends on the cell of origin, as well as the physiological status of the host^8^,^9^. In the case of infectious diseases, exosomes represent a source of biomarkers at two different levels: first, exosomes can carry pathogen associated molecular patterns^13^ and second, host proteins in the exosomes change as a result of an infectious process. In the first scenario, several studies have demonstrated that exosomes released from *Mtb*-infected cells contain mycobacterial products including proteins, RNAs and glycolipid^14^,^15^,^16^. More importantly, our laboratory recently published the discovery of a panel of *Mtb* peptides that were identified in exosomes isolated from the sera of TB patients and LTBI individuals, confirming the fact that *Mtb* infection modifies exosome composition and could be a source of biomarkers for TB^17^.

Regarding the host proteome, little is known about alterations in the exosome content influenced by *Mtb* infection. Taking into account that *Mtb* affects the protein composition of several organelles in infected cells (i.e. mitochondria, endoplasmic reticulum and the phagosome)^4^,^18^,^19^, and the intrinsic features of exosomes previously described, it is very likely that *Mtb* affects the composition of the human proteins loaded in exosomes of infected cells. Here, we aimed to identify significant changes in human proteins of exosomes derived from *Mtb* infected cells. Considering that protein localization within the exosome can play an important role in function of these vesicles, we implemented a novel biotinylation scheme to differentiate the surface-exposed proteins within the exosome membrane from those protected within the vesicle. This work serves as a proof-of-concept that intracellular *Mtb* affects the protein composition of exosomes released from host cells. In view of that, the host protein composition of exosomes from individuals infected with *Mtb* could aid in our understanding of the host-*Mtb* interaction and further TB biomarker efforts.

## Results and Discussion

### General characterization of exosomes is stable regardless of infection status

**Figure 1:**
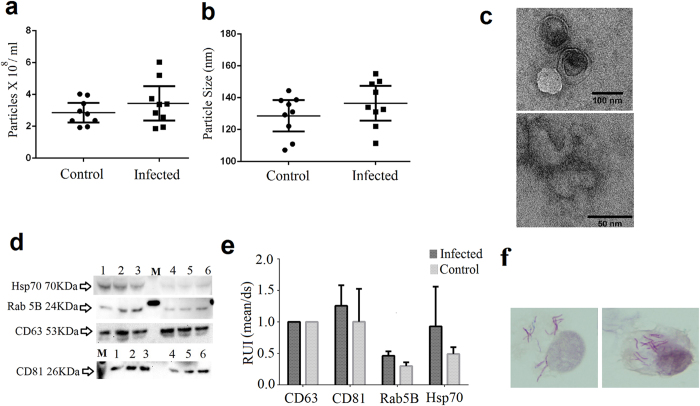
Characterization and validation of the human macrophages derived exosomes. Vesicle concentration (**a**) and size (**b**) from *Mtb-*infected and uninfected control cells. Differences between infected and control were not statistically different as determined by t-test, p = 0.299 and p = 0.236, respectively. More detailed results from the light scattering analysis are presented in the Supplementary Fig S1. Values represent three independent experiments and three technical replicates. Exosomes from *Mtb-*infected cells (**c**), upper picture) and from control cells (**c**), lower picture) viewed using transmission electron microscopy. Western blot (**d**) and densitometry analysis (**e**) of exosome hallmark proteins from three independent experiments. Exosomes from infected cells, lanes: 1, 2 and 3. M: Molecular weight marker. Exosomes from control cells lanes: 4, 5 and 6. The original images of the western blots can be found in Supplementary Fig. S2. THP-1 macrophages infected with *Mtb* (**f**) stained after exosome collection 100X magnification (Modified Kinyoun and hematoxylin staining). RUI: relative units of intensity.

The nanovesicle concentration when normalized by total protein was similar between samples, ranging from 2 × 10^8^ to 6 × 10^8^ particles per ml (t-test, p = 0.299). Exosomes from infected macrophages (MΦ) appeared to be slightly larger; however, the size difference was not significant (t-test, p = 0.236) (Fig. 1a/b). In addition to size and concentration, we detected the presence of CD63, CD81, and Rab-5B, considered exosome hallmark proteins, by western blot in all biological replicates (Fig. 1d/e). Abundance of these proteins was invariant amongst all samples. Finally, exosomes were visualized with transmission electron microscopy (Fig. 1c). These findings confirmed that the nanovesicles in this study were consistent with exosomes.

To determine the viability of *Mtb-*infected cells and controls cells after exosome collection, we did an assay based on the reducing capacity of viable cells using resaruzin (AlamarBlue-Thermo Scientific), a non-toxic permeable substrate, which once in the cytoplasm is reduced to resorufin. In general, the proportion of viable infected cells relative to their corresponding control ranged from 92% to 114% confirming the sufficient viability of the cells.

Finally, we confirmed the extent of bacterial infection at the final point of the experiment microscopically (Fig. 1f) and by bacterial enumeration. The average number of colony forming units (CFUs) was 1.05 × 10^7^ ± 5 × 10^6^ CFU/ml. This average represents the 33.8% resident bacilli from the initial bacterial inoculum. Both results allowed us to confirm that the isolated exosomes were produced from *Mtb-*infected cells.

### Proteome of exosomes released from MΦ infected with *Mtb*

**Figure 2:**
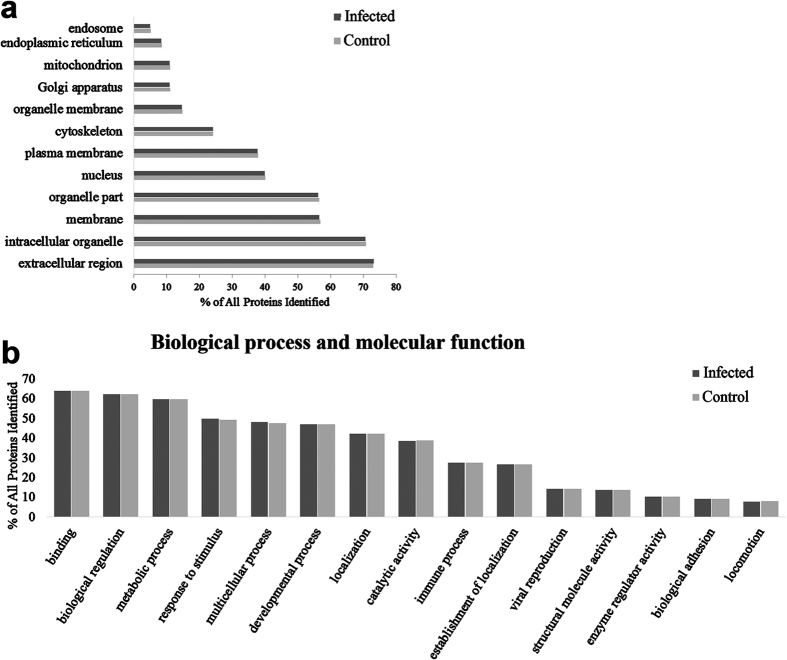
Function and localization of the proteins found in exosomes released from *Mtb-*infected macrophages and uninfected control cells. (**a**) Subcellular localization of most of the proteins found in the macrophage-derived exosomes. (**b**) The top 15 identified molecular functions and biological process associated with the proteins found in exosomal samples. Information obtained directly from the NCBI database.

Here we describe for the first time the human proteome of exosomes released from MΦ infected with *Mtb.* Overall we identified 355 proteins, from which 201 were classified as membrane associated proteins based on the GO term annotations from the NCBI database (Fig. 2a; Supplementary Table S1). Twenty-eight percent of the identified proteins were exclusively present in exosomes from infected MΦ.

Most of the proteins found in our exosome samples are involved in binding, immunological and metabolic processes (Fig. 2b). All of these processes have been previously associated with potential roles for exosomes^20^,^21^,^22^.

### Comparative proteomic analysis reveals significant differences between exosomes from infected and control cells

**Figure 3:**
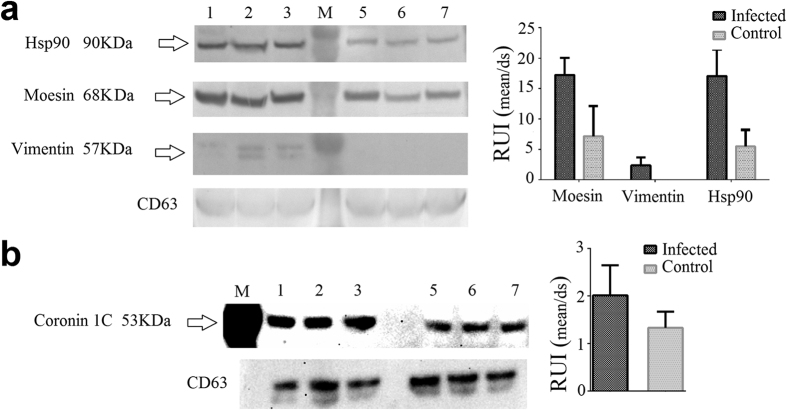
Western blot confirmed proteins significantly more abundant in exosomes from *Mtb-*infected cells originally detected by LC-MS/MS. Hsp90, Moesin and Vimentin were detected using a chromogenic substrate (**a**). The intensity of the bands was evaluated relative to the intensity of CD63. Additionally, Coronin 1 C was detected using a chemiluminescent substrate (**b**). The intensity of the bands was evaluated relative to the intensity of CD63. Samples from three independent experiments were evaluated. Exosomes from infected cells, lanes: 1, 2 and 3. M: Molecular weight marker. Exosomes from control cells lanes: 5, 6 and 7. The original images of the western blots can be found in Supplementary Fig. S3. RUI: relative units of intensity.

As we predicted, the infection of MΦ with *Mtb* impacted the protein composition of exosomes. To analyze the difference in protein abundance between exosomes from *Mtb-*infected and control cells, we used normalized spectral abundance factor (NSAF)^23^ to accurately compare individual protein abundances among our two samples. Forty-one proteins were significantly more abundant in exosomes in *Mtb-*infected cells (Table 1), a subset of these were confirmed by western blot (Fig. 3), including: HSP90, vimentin, Coronin 1 C and moesin. Previous studies have shown that some of these proteins play important roles during *Mtb* infection. Shekhawat *et al*., showed that human HSP proteins (including HSP90) were increased in sera from individuals with a high risk of being latently infected with *Mtb,* suggesting HSP proteins as potential biomarkers for LTBI^24^. We observed that HSP90 was significantly higher in exosomes from infected cells. In this study, we also found that infected MΦ produced exosomes with a higher concentration of vimentin compared with control cells. Vimentin is a ligand for NKp46, a receptor in Natural Killer cells (NK). The interaction between NKp46 and vimentin mediates the lysis of *Mtb-*infected cells by NK^25^. Exosomes enriched with vimentin could interfere in the interaction of NKp46 with the cell membrane associated vimentin (from MΦ), thus delaying the killing of *Mtb* infected cells. In this way, intracellular mycobacteria could be modulating the loading of vimentin in exosomes as a defense mechanism. Further investigations into this phenomenon could give us new insights about the complex host-pathogen interaction in TB. Lastly, L-amino acid oxidase (LAAO) was more abundant in exosomes from *Mtb*-infected MΦ in our study. This protein plays important roles in the innate immune response acting as an antibacterial enzyme that catabolizes the deamination of L-amino acids producing H_2_O_2_ and ammonia^26^. Our results suggest that *Mtb-*infected cells could be using exosomes to export endogenous antimicrobial molecules as a defense mechanism. This phenomenon has been previously observed with IFNα that was transported between cells via exosomes^20^. Collectively, these changes in exosomes as a consequence of *Mtb* infection could reveal important information regarding the host-pathogen interaction.

### Significant changes of the exosome membrane proteome after infection with *Mtb*

Exosomes are an important source of biomarkers for TB by virtue of the discovery of mycobacterial molecules packaged within these vesicles^14^,^15^,^17^,^27^. Here, the effect of the infection on host exosomal protein composition illustrates another potential opportunity to exploit exosomes as biomarkers of TB. In this regard, we sought to explore the differential abundance of membrane associated proteins since they represent a more accessible set of targets for downstream development of a biomarker assay. Using GO-term annotation analysis, we found that 63% (26/41) of the proteins that were significantly more abundant in exosomes from infected cells were also membrane associated. To better characterize this subset of proteins we used transmembrane helix prediction software and found that 31% of the 26 proteins contained at least one probable transmembrane domain or residues that were potentially membrane associated (Table 2).

To further ascertain the localization of the proteins that were significantly more abundant in exosomes from infected cells, we conducted an experiment to differentially label proteins based on their localization within the exosome. First, intact exosomes were exposed to NHS-Sulfo-LC-LC-Biotin, a negatively charged molecule unable to permeate biological membranes, to label proteins that were exclusively facing-out of the exosomal membrane. NHS-Sulfo-LC-LC-Biotin binds amino-terminus and lysine residues, increasing the molecular mass of the labeled peptide by 452 Da. The fact that this molecule has a spacer arm of 30.5 Å improves its accessibility to folded proteins in the membrane of intact exosomes. After cleaning the excess of NHS-Sulfo-LC-LC-Biotin, the biotinylated exosomes were lysed followed by secondary labeling of proteins on the internal-face of the exosomal membrane or within the lumen of the exosome, using an alternate biotin reagent (Sulfo-NHS-Biotin) that increases the molecular weight of the labelled residues by 226 Da. This labeling strategy allowed for the identification and differentiation of protein populations by mass spectrometry analysis, including determination of protein domains exposed to the external side of the exosome.

Our labeling studies demonstrated that 6 of 26 differentially abundant membrane proteins identified by GO-term analysis were differentially labeled with LC-LC biotin, and thus are surface exposed (Table 2). In addition, this technique afforded the identification of one additional surface exposed protein, a nucleoside diphosphate kinase (Ndk) (Table 3). Interestingly, *Mycobacterium*-derived Ndk has been associated with a greater survival of infected macrophages^28^. Ndk metabolizes extracellular ATP which plays important roles during the inflammatory response and macrophage activation via P2X_7_ receptor^29^. Although human derived-Ndk has been mostly related with intracellular vesicle trafficking^29^, human-derived Ndk in secreted exosomes could be acting as an ecto-enzyme that modulates macrophage activation and survival in an ATP-dependent manner; favoring mycobacterial persistence. Three of the seven LC-LC biotin labeled proteins (Table 3) were also labeled with the shorter biotin (226 Da label) (Table 2); these are indicative of transmembrane proteins with both external and internal segments. In the same way, the presence of proteins exhibiting only the small biotin implies that the protein is confined to the exosome lumen. Our biotinylation strategy represents an original approach that allows for a more specific characterization of exosomes.

## Conclusions

Our study demonstrates that the infection with *Mtb* influences changes in the protein composition of exosomes released from infected cells. Even though our findings do not have an immediate translational application they represent the proof-of-concept that *Mtb-*infected cells will produce exosomes with a characteristic proteome. The confirmation of these phenomena in a clinically relevant sample set (i.e. TB-patient sera derived exosomes) will advance our knowledge about *Mtb-*host interactions and will offer a potential source for new TB biomarkers.

## Methods

### Human monocytes growth and activation

THP-1 human monocytes (American Type Culture Collection, ATCC/TIB-202) were cultured at 37 °C at 5% CO_2_ in complete RPMI (cRPMI) media. This media contained RPMI 1640 base medium (ATCC) supplemented with 2-mercaptoethanol (Gibco) (final concentration of 0.05 mM) and 10% exosome-depleted fetal bovine serum (EXO-FBS, SBI). Monocytes (2.5 × 10^5^ cells/ml) were activated to macrophages (MΦ) using 200 nM of Phorbol 12-myristate 13-acetate (PMA) (Sigma-Aldrich) for 72 h. After the activation, unbound cells and remaining media were removed, and the adherent cells were washed three times using phosphate buffer saline (PBS).

### Macrophage infection

All procedures involving live *Mtb* were completed in a biosafety level 3 laboratory at Colorado State University. Infectivity stocks of *Mtb* strain H37Rv were thawed and spun down at 1,200 × *g* for 10 minutes. The pellet was suspended in cRPMI and bath-sonicated for 1 minute to disrupt bacteria clumps. THP-1 MΦ were infected with *Mtb* at a 1:5 ratio (MΦ: Bacteria), for 4 h at 37 °C/5% CO_2_. After infection, the THP-1 MΦs were washed three times with PBS and then 25 ml of fresh cRPMI was added. The cells were returned to culture conditions for 24 h for the production of exosomes. Identical flasks of THP-1 MΦ were treated in the same way but without *Mtb* to produce control exosomes. Every experiment with *Mtb* infected and uninfected THP-1 MΦ was done in triplicate and three independent experiments at different days were done as biological replicates.

### Exosome purification

Approximately, 25 ml of supernatant from infected and control THP-1 MΦ were collected after 24 h of infection and filtered through 0.2 μm membrane to remove cellular debris, potential membrane fragments from lysed cells, large vesicles (>500 nm) and whole bacteria. The collected material was filtered using an Amicon centrifugal filter unit with a molecular weight cut-off (MWCO) of 100 KDa (EMD Millipore) to 2 ml concentration to remove most of the soluble proteins. The exosome-rich retentate was diluted to 15 ml with PBS and filtered again through the 100 KDa to elute remaining soluble proteins. After this, the retained sample was centrifuged at 18,000 × *g* for 30 min to pellet larger vesicles. The resultant supernatant was mixed with 400 μl of ExoQuick-TC (SBI) and incubated at 4 °C overnight to precipitate exosomes. After this, the mix was centrifuged at 2,600 × *g* for 30 min. The pellet-containing exosomes was suspended in 1 ml of PBS and the total protein concentration was measured using the microbicinchoninic acid assay (microBCA, Thermo Scientific). The exosomes were aliquoted (50 μg/vial) and stored at −20 °C until further analysis.

### THP-1 MΦ viability test after *Mtb* infection

To guarantee the accurate comparison between exosomes from *Mtb-*infected and control cells, the viability of both types of cells was evaluated after exosome collection. Briefly, after each experiment, the cells were washed with PBS and detached from the flask using 5 ml of 0.25% trypsin-EDTA (Gibco) at 25 °C for 10 min. Five ml of cRPMI were added and the cells were centrifuged at 600 × *g* per 10 min and washed with cRPMI once. Then, cells were suspended in cRPMI to perform the Alamar Blue (Invitrogen) viability assay. Briefly, 100 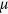
l of previously suspended control and infected cells were added to a 96 wells plate in triplicate. Subsequently, 10 μl of Alamar Blue reagent was added to each well and the plate was incubated at 37 °C/5%CO_2_ for 4 h in the dark. The viability of the infected and control cells was determined following the manufacturer’s recommendations.

### Validation of *Mtb* infection following exosome purification

Ten μl of infected cells were pipetted onto a microscope slide, fixed with paraformaldehyde 4% for 24 h and stained by the Kinyoun acid-fast procedure and microscopically evaluated. Then, the remaining infected THP-1 MΦ were pelleted and lysed using 0.05% SDS for 3 min. The SDS treatment was enough to lyse the THP-1 MΦ but not the intracellular bacteria. The suspension was centrifuged at 1,200 × *g* for 10 min and the pellet was suspended in Middlebrook 7H9 broth. Seven 10-fold serial dilutions were plated on 7H11 quad-plates and incubated at 37 °C for 3 weeks for colony forming units (CFU) enumeration to verify the number of infecting bacteria after exosome production.

### Characterization of exosomes

The concentration and size of the exosomes for all samples were evaluated by nanoparticle tracking analysis (NTA), using the NanoSight NS300 (Malvern Instruments). From each exosome-sample, 5 μg of total protein were diluted in 1 ml of PBS and loaded into the instrument. Each sample was analyzed in triplicate. The presence of hallmark exosome proteins was evaluated by Western Blot.

### Western Blot (WB) analysis

Exosome samples (50 ug) from three independent experiments were resolved in a NuPAGE Novex 4–12% Bis-Tris Gel (Life Technologies) and transferred to a nitrocellulose membrane, 0.2 μm (BIO-RAD). Then, the membrane was blocked with bovine serum albumin 2% for 1 h. After this, the primary antibody was added and incubated during 1 h. Subsequently, the conjugated antibody was added and finally the membrane was exposed to the developer reagent. For exosome characterization the primary antibodies: anti-CD63 (SBI), anti-CD81 (SBI) and anti-Rab5B (A-20) (Santa Cruz Biotechnology) were used. For validation of the proteomics results, the primary antibodies: anti-Coronin 1 C (G-R2) (Santa Cruz Biotechnology), anti-Moesin (Life Technologies), anti-Vimentin Antibody (9E7E7) (Santa Cruz Biotechnology) and anti-HSP 90 (F8) (Santa Cruz Biotechnology) were used. Two different HRP-conjugated antibodies were used: Goat Anti-Rabbit F(ab)2 fragment (Thermo Scientific) and Goat anti-Mouse IgG (H + L) (Thermo Scientific) depending on the source of the primary antibody. For the WB detecting moesin and HSP 90, the colorimetric substrate 4-Chloro-1-Naphtol (Sigma) was used. For the analysis of the other proteins the chemiluminescent substrate Super signal West Pico (Thermo Scientific) was used. WB images and band intensities were determined using the Chemi-Doc XRS+ with Image Lab software version 3.0 (BIORAD). For some of the assays, after transferring the protein to the nitrocellulose membrane, a Ponceau S stain was done to verify the efficiency of the transfer (Supplementary Fig. S3). For this, the membrane was soaked in Ponceau S solution (Ponceau S 0.2%, Acetic acid 3% in distilled water) for 5 minutes, washed with distilled water for approximately 5 minutes to remove unspecific staining and a scan of the membrane was saved. After this, the membrane was washed with PBS 1X for 5 minutes, 3 times. Then, the western blot procedure was followed as described above.

### Transmission electron microscopy

For electron microscopy analysis, exosome samples from *Mtb-*infected and control cells were prepared following the protocol described by Théry *et al*.^30^. Briefly, exosome samples were mixed with paraformaldehyde 4% (1:1 ratio) and incubated overnight at 4 °C. Then, a formvar/carbon coated grid (Electron Microscopy Science) was placed on top of a drop of 10 ul of exosome preparation for 20 min. Then, the grid was transferred to a drop of 100 ul of PBS, and then, transfer to a 50 ul drop of glutaraldehyde 1% for 5 min. After this, the grid was washed 7 times with 100 ul drops of distilled water. Next, the grid was transferred to a 50 ul drop of uranyl-oxalate for 5 min. Finally, the grid was transferred to a 50 ul drop of methylcellulose/uranyl acetate (9:1 ratio) for 10 min on ice. After this, excess of methylcellulose/uranyl acetate was removed by blotting on Whatman #1. The grids were air dried and observed in the transmission electron microscope JEOL 2100 F at 200 kV.

### Biotinylation of exosome proteins

Two different types of biotin reagents were used in this experiment. Sulfo-NHS-Biotin (Thermo Scientific), containing a shorter, 13.5 Å spacer arm biotin, was used to label the proteins in the lumen or in the internal leaflet of the exosomal membrane and Sulfo-NHS-LC-LC-Biotin (Thermo Scientific), was used to label the proteins exposed to the external leaflet of intact exosomes and contains a larger, 30.5 Å spacer arm between the biotin and amine reactive linker—the size of this linker helps to overcome steric hindrance and increases labeling efficiency at the crowded exosome surface. Two hundred micrograms of intact exosomes were mixed with 10 mM Sulfo-NHS-LC-LC-Biotin at room temperature for 30 min. Four conditions were taken into account during this experiment: (*a*) an excess of Sulfo-NHS-LC-LC-Biotin was used to favor a complete saturation of exposed lysine residues and potential N-terminus, (*b*) the presence of the sulfonate group in Sulfo-NHS-LC-LC-Biotin blocks the reagent from penetrating the exosomal membrane, (*c*) Sulfo-NHS-LC-LC-Biotin has an spacer arm of 30.5 angstroms which improves the biotinylation of proteins in their natural conformation, and (*d*) amino acids labeled with Sulfo-NHS-LC-LC-Biotin will have an increase in mass of 452 Da. After incubation, the excess of Sulfo-NHS-LC-LC-Biotin was removed using a 10 KDa MWCO filtration device. Biotinylated exosomes were washed with 10X volumes of 1X PBS and concentrated to a final volume of 30 μl. Biotinylated exosomes were lysed with 300 ul of RIPA buffer (Thermo Scientific) for 1 h, followed by three freeze and thaw cycles. After lysis, buffer exchange was done to replace RIPA buffer with 1X PBS. RIPA buffer contains primary amines that interfere with the next biotinylation step. Lysed-biotinylated exosomes were exposed to 10 mM Sulfo-NHS-Biotin (Thermo Scientific), at room temperature for 30 min, for labeling of remaining free amines. Excess of biotin was removed as mentioned above.

### Protein digestion

#### Two strategies were applied based on exosome preparation (biotinylated or unlabeled)

##### Unlabeled samples

50 μg of exosomes were mixed with Laemmli SDS-PAGE buffer which contains: 2-Mercaptoethanol (5%), Bromophenol blue (0.01%), Glycerol (50%), SDS (electrophoresis-grade, 2%), and Tris-HCl, 63 mM (pH 6.8). Then, samples were heated at 100 °C for 5 minutes. After this, the samples were resolved in a NuPAGE Novex 4–12% Bis-Tris Gel (Life Technologies) for 5 min. Then, the gels were stained with Coomassie Blue (Invitrogen) for 1 h to visualize the localization of the proteins and destained briefly in water to clarify protein bands. Afterwards, the entire lane of gel containing the proteins was excised and cut into 1 mm^3^ pieces that were transferred to an Eppendorf tube and mixed with destaining solution (60% acetonitrile (ACN) in ammonium bicarbonate 0.2 M) for 1 hour at 37 °C, twice. After destaining, the gel pieces were vacuum dried. Dried gel pieces were mixed with trypsin (Roche) in 0.2 M ammonium bicarbonate at a ratio of 50:1 (w/w-sample: trypsin) at 37 °C, overnight. The next day, the tryptic peptides were extracted by adding 100 μl of 60% ACN, 0.1% trifluoroacetic acid in HPLC-grade water and incubated at 37 °C for 1 hour, twice. The extracted peptides were vacuum dried, suspended in Solvent A (3% ACN and 0.1% formic acid in HPLC-grade water), centrifuged at 13,000 × *g* for 5 min to remove debris, solution transferred into auto-sampler vials (Agilent technologies), and samples stored at −20 °C in until analysis by LC-MS/MS.

##### Biotinylated samples

50 μg of labeled exosome lysates were subject to gel electrophoresis and staining as described above. The biotin label binds to the free amine group of lysine residues, thus, interfering with one of the cleavage sites for trypsin; as an alternative, the endoproteinase AspN (Roche) was used for protein digestion. After obtaining the dried and destained gel pieces as described above, protein samples were reduced by incubation with 5 mM dithiothreitol (Sigma) for 20 min at 50 °C and alkylated by incubation with 15 mM iodoacetamide (Sigma) at 25 °C for 15 min in the dark, following the AspN manufacturer’s recommendations. Proteins were then digested with AspN in 0.2 M ammonium bicarbonate using a 50:1 ratio (w/w-sample: enzyme). Subsequent steps after enzymatic digestion were similarly performed as described for trypsin digestion.

### Liquid chromatography-tandem mass spectrometry (LC-MS/MS)

Approximately 1 μg of digested peptides for each sample was injected using an EASY nanoLC-II system (Thermo Scientific, San Jose, CA). Peptides were purified and concentrated using an on-line enrichment column (EASY-Column, 100 μm ID × 2 cm ReproSil-Pur C18). Subsequent chromatographic separation was performed on a reverse phase nanospray column (EASY-Column, 3 μm, 75 μm ID × 100 mm ReproSil-Pur C18) using a 90 minute linear gradient from 5–45% solvent B (100% ACN, 0.1% formic acid) at a flow rate of 400 nanoliters/min. Peptides were eluted directly into the mass spectrometer (Thermo Scientific Orbitrap Velos). The instrument was operated in Orbitrap-LTQ mode where precursor measurements were acquired in the Orbitrap (60,000 resolution) and MS/MS spectra (top 20) were acquired in the LTQ ion trap with a normalized collision energy of 35%. Mass spectra were collected over a m/z range of 400–2000 Da using a dynamic exclusion limit of 2 MS/MS spectra of a given peptide mass for 30 s (exclusion duration of 90 s). Compound lists of the resulting spectra were generated using Xcalibur 2.2 software (Thermo Scientific) with an S/N threshold of 1.5 and 1 scan/group.

### Data Analysis

Tandem mass spectra were extracted, charge state deconvoluted and deisotoped by ProteoWizard (MSConvert version 3.0). Raw data files were converted to mzXML format and submitted to the Sorcerer2 integrated data analysis platform (Sage-N Research, version 5.0.1); subsequent MS/MS analysis was performed using SEQUEST (Sage-N Research, Milpitas, CA, USA; version v. 3.5). SEQUEST was set up to search the Uni-Prot *Homo sapiens* Reference Proteome (ID: UP000005640, 70076 entries) assuming the enzymatic digestion with trypsin (after Arg or Lys) or AspN (before Asp or Glu) depending on which enzyme was used. SEQUEST was searched with a fragment ion mass tolerance of 1.00 Da and a parent ion tolerance of 50 PPM. Oxidation of methionine (+15.99) and carbamidomethyl of cysteine (+57.02) were specified in SEQUEST as variable modifications for the unlabeled experiment. Biotin (+226) and LC-LC Biotin (+452) in lysine and N-termini were also included as variable modification for the labeling experiment.

Scaffold (version Scaffold_4.5.1, Proteome Software Inc., Portland, OR) was used to validate MS/MS based peptide and protein identifications. Peptide identification thresholds were set such that a peptide FDR of 1% and a peptide confidence threshold of 95% was achieved based on hits to the reverse database^31^. Protein identifications were accepted if they could be established at greater than 99.0% probability to achieve an FDR less than 1.0% and contained at least 2 identified peptides. Protein probabilities were assigned by the Protein Prophet algorithm^32^. Proteins that contained similar peptides and could not be differentiated based on MS/MS analysis alone were grouped to satisfy the principles of parsimony. Proteins were annotated with GO terms from NCBI (downloaded Dec 31, 2015)^33^. The mass spectrometry proteomics data have been deposited to the ProteomeXchange Consortium (http://proteomecentral.proteomexchange.org) via the PRIDE partner repository^34^ with the dataset identifier PXD004062 and DOI 10.6019/PXD004062. Membrane associated proteins were also explored using the free online software TMHMM (Version 2.0)^35^,^36^.

Differences in protein abundances between exosomes from *Mtb*-infected MΦ versus control cells were evaluated by *t*-test, using normalized spectral abundance factor (NSAF)^23^. P values < 0.05 were accepted as statistically significant. For validation of the proteomics results a subset of proteins that were significantly higher in exosomes from infected cells were evaluated by western blot as described above.

## Additional Information

**How to cite this article**: Diaz, G. *et al*. Changes in the Membrane-Associated Proteins of Exosomes Released from Human Macrophages after *Mycobacterium tuberculosis* Infection. *Sci. Rep.* **6**, 37975; doi: 10.1038/srep37975 (2016).

**Publisher's note:** Springer Nature remains neutral with regard to jurisdictional claims in published maps and institutional affiliations.

## Supplementary Material

Supplementary Comprehensive Data and Information

## Figures and Tables

**Table 1:** Proteins significantly different between exosomes from *Mtb*-infected and control macrophages.

Identified Proteins	Infected NSAF	Control NSAF	p value NSAF-inf versus NSAF-control	Fold Change NSAF-inf/NSAF-control
60 S acidic ribosomal protein P0	0.064	0.000	0.00012	INF
Coronin-1C	0.023	0.000	0.00017	INF
Lupus La protein	0.023	0.000	0.00019	INF
Heterogeneous nuclear ribonucleoprotein K	0.075	0.003	0.00029	28.4
Heat shock 70 kDa protein 4	0.013	0.000	0.00031	INF
Alanine-tRNA ligase, cytoplasmic	0.006	0.000	0.00035	INF
Calreticulin	0.017	0.000	0.001	INF
Protein disulfide-isomerase A3	0.040	0.000	0.002	INF
L-amino-acid oxidase	0.018	0.000	0.003	INF
Moesin	0.151	0.062	0.0032	2.4
Nucleolin	0.063	0.007	0.0032	8.6
Vimentin	0.251	0.072	0.0034	3.5
Protein disulfide-isomerase A6	0.046	0.003	0.0035	16.6
Spliceosome RNA helicase DDX39B	0.027	0.000	0.0039	INF
Fermitin family homolog 3	0.046	0.002	0.0047	19.8
Programmed cell death 6-interacting protein	0.005	0.000	0.0047	INF
S-adenosylmethionine synthase isoform type-2	0.029	0.000	0.0048	INF
Glyceraldehyde-3-phosphate dehydrogenase	0.293	0.201	0.0059	1.5
ATP-dependent RNA helicase A	0.005	0.000	0.0068	INF
60 kDa heat shock protein, mitochondrial	0.013	0.000	0.0082	INF
Cytosol aminopeptidase	0.041	0.000	0.0084	INF
Ubiquitin-like modifier-activating enzyme 1	0.056	0.007	0.0089	8.5
ITIH4 protein	0.011	0.000	0.01	INF
Serine/threonine-protein phosphatase 2 A 65 kDa regulatory subunit A alpha isoform	0.011	0.002	0.011	7.0
Tryptophan-tRNA ligase, cytoplasmic	0.031	0.000	0.011	INF
Transketolase	0.082	0.015	0.012	5.6
Zyxin (Fragment)	0.007	0.000	0.012	INF
Heat shock protein HSP 90-beta	0.361	0.221	0.014	1.6
Tyrosine-tRNA ligase, cytoplasmic	0.014	0.000	0.017	INF
6-phosphogluconate dehydrogenase, decarboxylating	0.075	0.021	0.024	3.6
X-ray repair cross-complementing protein 6	0.061	0.004	0.026	13.8
78 kDa glucose-regulated protein	0.109	0.047	0.028	2.3
Eukaryotic initiation factor 4A-I	0.115	0.038	0.028	3.1
Thrombospondin-4	0.011	0.002	0.028	6.2
Bifunctional purine biosynthesis protein PURH	0.028	0.000	0.028	INF
Staphylococcal nuclease domain-containing protein 1	0.012	0.001	0.031	9.2
Heat shock cognate 71 kDa protein	0.443	0.273	0.036	1.6
Integrin beta-1	0.006	0.000	0.046	INF
UDP-glucose 6-dehydrogenase	0.013	0.000	0.046	INF
Purine nucleoside phosphorylase	0.039	0.000	0.048	INF
Lamin-B1	0.021	0.003	0.049	6.5
Transforming growth factor-beta-induced protein ig-h3	0.061	0.128	0.0047	0.5
Palmitoyl-protein thioesterase 1	0.053	0.106	0.033	0.5
Complement C4-A	0.056	0.073	0.035	0.8

INF: When the value in the denominator is zero (the fold change is the normalized spectral abundance factor^23^ (NSAF)-infected divided by NSAF-control), ^*^p value from *t*-test comparing the averages of three independent experiments between NSAF-infected versus NSAF-control.

**Table 2:** Membrane associated proteins significantly more abundant in exosomes from *Mtb*-Infected cells and their biotinylation pattern.

Membrane associated protein‡	Proteins with AA residues in transmembrane domains**	LC-LC Biotin	Biotin
60S acidic ribosomal protein P0	X		
Coronin-1C	X		
Heterogeneous nuclear ribonucleoprotein K			
Alanine-tRNA ligase, cytoplasmic			
Calreticulin	X		
Protein disulfide-isomerase A3	X		
Moesin		X	
Nucleolin			
Vimentin		X	X
Protein disulfide-isomerase A6	X		
Fermitin family homolog 3			
Programmed cell death 6-interacting protein			
Glyceraldehyde-3-phosphate dehydrogenase		X	X
ATP-dependent RNA helicase A	X		
60 kDa heat shock protein, mitochondrial			
Cytosol aminopeptidase			
Serine/threonine-protein phosphatase 2A 65 kDa regulatory subunit A alpha isoform			
Transketolase	X		
Heat shock protein HSP 90-beta		X	
78 kDa glucose-regulated protein			
Eukaryotic initiation factor 4A-I		X	X
Bifunctional purine biosynthesis protein PURH			
Staphylococcal nuclease domain-containing protein 1			
Heat shock cognate 71 kDa protein		X	
Integrin beta-1	X		
Lamin-B1			

^‡^Classification based on the Go-term annotations from the NCBI database.

^**^Based on TMHMM Server v. 2.0 prediction of transmembrane helices in proteins (AA: Aminoacids).

**Table 3:** List of proteins and the specific peptide(s) labelled with LC-LC biotin.

Protein name	Peptide sequence
Eukaryotic initiation factor 4AI	EVQkLQMEAPHIIVGTPGRVF
Glyceraldehyde-3-phosphate dehydrogenase	DNFGIVEGLMTTVHAITATQkTV
Heat shock cognate 71 kDa protein	DPVEkALR
Heat shock protein HSP 90-beta	ERIMkAQALR
Moesin	EEAkEALLQASR
Nucleoside diphosphate kinase	ERTFIAIkP
Vimentin	DVRQQYESVAAkNLQEA

k: designates a lysine residue with the LC-LC biotin modification.
