# Supplementary material for: Changes in the Membrane-Associated Proteins of Exosomes Released from Human Macrophages after Mycobacterium tuberculosis Infection
Source: Sci Rep. 2016 Nov 29;6:37975. doi: 10.1038/srep37975 (PMC5126699; doi:10.1038/srep37975)
Supplement: Supplementary Comprehensive Data and Information [file srep37975-s1.pdf]

Changes in the Membrane-Associated Proteins of Exosomes Released from Human Macrophages after *Mycobacterium tuberculosis* Infection.

Gustavo Diaz[1], Lisa M. Wolfe[2], Nicole A. Kruh-Garcia[1], and Karen M. Dobos[1*].

[1]Department of Microbiology, Immunology and Pathology, Colorado State University, Fort Collins, Colorado, United States of America

[2]Proteomics and Metabolomics Facility, Colorado State University, Fort Collins, Colorado, United States of America

* Corresponding author

Karen M. Dobos.

E-mail: Karen.Dobos@colostate.edu. Phone: 1+970-491-1891/970-491-2229. Fax: 1+970- 491-1815

1682 Campus delivery, Microbiology, Immunology and Pathology Department. Colorado State University, Fort Collins. Colorado. United States of America.

Supplementary table S1: List of all proteins identified in exosomes from *Mtb* infected and control cells. Protein identifications were accepted if they could be established at greater than 99.0% probability to achieve an FDR less than 1.0% and contained at least 2 identified peptides.

Supplementary table S1: List of all proteins identified in exosomes from *Mtb* infected and control cells

				NORMALIZED SPECTRAL ABUNDANCE FACTOR						TOTAL SPECTRUM COUNT						EXCLUSIVE UNIQUE SPECTRUM COUNT						PERCENTAGE OF COVERAGE					
#	Identified Proteins (355)	Accession Number	Molecular Weight	Control 1	Control 2	Control 3	Infected 1	Infected 2	Infected 3	Control 1	Control 2	Control 3	Infected 1	Infected 2	Infected 3	Control 1	Control 2	Control 3	Infected 1	Infected 2	Infected 3	Control 1	Control 2	Control 3	Infected 1	Infected 2	Infected 3
1	Alpha-2-macroglobulin	A2MG_HUMAN	163 kDa	0.28	0.49	0.50	0.26	0.23	0.28	445	459	483	514	378	462	29	16	20	24	15	19	20.80%	10.70%	13.80%	14.00%	10.60%	13.00%
2	Keratin, type I cytoskeletal 10	K1C10_HUMAN	59 kDa	0.85	0.87	0.37	0.42	0.41	0.49	540	323	142	334	272	320	38	32	23	34	32	35	42.30%	39.60%	33.20%	40.90%	40.10%	42.30%
3	Complement C3	CO3_HUMAN	187 kDa	0.15	0.36	0.28	0.11	0.20	0.18	266	385	310	250	370	334	15	16	12	16	20	14	8.00%	8.66%	5.23%	7.40%	11.00%	5.83%
4	Fibronectin	FINC_HUMAN	263 kDa	0.10	0.22	0.14	0.07	0.15	0.10	250	329	222	233	400	276	39	43	32	37	57	44	23.30%	23.90%	18.50%	19.30%	29.50%	25.10%
5	Keratin, type II cytoskeletal 1	K2C1_HUMAN	66 kDa	0.63	0.68	0.42	0.37	0.45	0.44	462	293	191	322	328	327	41	29	24	38	33	38	49.70%	40.70%	40.20%	49.40%	50.50%	50.50%
6	Gelsolin	GELS_HUMAN	86 kDa	0.19	0.51	0.50	0.40	0.40	0.40	159	255	257	429	356	347	21	18	19	28	27	27	41.80%	32.70%	35.00%	50.80%	48.20%	47.20%
7	Talin-1	TLN1_HUMAN	270 kDa	0.10	0.13	0.12	0.12	0.16	0.17	280	205	206	413	448	497	50	33	37	69	69	73	29.10%	19.30%	20.00%	36.20%	37.20%	37.00%
8	Adenylyl cyclase-associated protein 1	CAP1_HUMAN	52 kDa	0.68	0.33	0.58	0.55	0.48	0.62	352	99	183	359	259	333	33	18	23	38	29	35	46.30%	36.60%	40.40%	46.30%	41.70%	46.70%
9	Actin, cytoplasmic 2	ACTG_HUMAN	42 kDa	0.61	0.78	0.78	0.60	0.68	0.50	247	185	194	308	288	212	23	19	21	26	25	23	50.10%	42.40%	46.40%	53.60%	55.70%	48.00%
10	Heat shock cognate 71 kDa protein	HSP7C_HUMAN	71 kDa	0.22	0.24	0.36	0.38	0.46	0.49	147	100	152	334	335	354	15	14	18	40	36	35	25.20%	24.00%	27.40%	44.90%	39.30%	44.70%
11	Heat shock protein HSP 90-beta	HS90B_HUMAN	83 kDa	0.17	0.25	0.25	0.32	0.38	0.38	98	82	89	249	241	239	22	14	22	36	37	31	25.70%	22.20%	26.70%	46.00%	39.60%	37.20%
12	Keratin, type II cytoskeletal 2 epidermal	K22E_HUMAN	65 kDa	0.60	0.53	0.23	0.37	0.24	0.37	419	215	98	323	171	266	47	32	16	29	27	32	70.70%	58.20%	39.60%	57.90%	53.10%	62.90%
13	Pyruvate kinase PKM	KPYM_HUMAN	58 kDa	0.45	0.45	0.45	0.46	0.55	0.50	257	152	158	332	331	298	22	17	18	37	27	27	38.40%	35.00%	35.00%	48.80%	37.90%	41.60%
14	Inter-alpha-trypsin inhibitor heavy chain H2	A0A087WTE1_HUMAN	107 kDa	0.05	0.30	0.25	0.09	0.21	0.16	56	179	153	114	226	169	10	13	14	11	14	14	9.31%	10.30%	10.30%	9.42%	10.30%	12.00%
15	Keratin, type I cytoskeletal 9	K1C9_HUMAN	62 kDa	0.55	0.34	0.26	0.32	0.44	0.30	369	133	105	270	309	209	27	11	11	20	21	17	45.70%	28.90%	37.40%	47.50%	43.50%	43.80%
16	Serum albumin	A0A0C4DGB6_HUMAN	69 kDa	0.17	0.33	0.29	0.10	0.22	0.15	110	128	116	80	147	105	3	3	3	3	4	4	2.48%	2.48%	2.48%	2.48%	3.81%	3.81%
17	Apolipoprotein E	APOE_HUMAN	36 kDa	0.52	1.11	0.85	0.30	0.54	0.47	180	223	178	131	193	168	21	26	22	18	21	24	52.70%	61.20%	57.40%	53.60%	55.80%	56.80%
18	Periostin	POSTN_HUMAN	93 kDa	0.06	0.29	0.27	0.13	0.19	0.21	57	152	149	150	179	195	7	11	13	16	15	17	9.21%	14.00%	17.20%	18.50%	17.30%	19.90%
19	Tubulin beta chain	TBB5_HUMAN	50 kDa	0.30	0.26	0.41	0.35	0.41	0.38	135	68	113	207	195	184	22	11	21	31	25	25	60.40%	31.30%	58.30%	67.60%	63.50%	60.10%
20	Complement C4-A	CO4A_HUMAN	193 kDa	0.06	0.08	0.08	0.05	0.06	0.06	123	86	89	117	112	121	11	10	11	10	10	10	4.42%	4.42%	4.42%	4.42%	4.42%	4.42%
21	Alpha-actinin-4	ACTN4_HUMAN	105 kDa	0.15	0.12	0.10	0.19	0.13	0.16	145	69	58	221	136	159	27	11	12	39	23	27	34.80%	17.60%	17.10%	46.40%	31.80%	33.80%
22	Alpha-enolase	ENOA_HUMAN	47 kDa	0.14	0.30	0.30	0.29	0.42	0.33	64	83	87	169	208	161	10	11	10	18	19	17	31.80%	28.60%	27.20%	42.60%	39.40%	39.40%
23	Coagulation factor V	A0A0A0MRJ7_HUMAN	252 kDa	0.06	0.04	0.04	0.05	0.02	0.03	142	57	59	147	50	72	13	5	6	15	9	9	4.80%	2.92%	2.92%	5.34%	3.54%	3.41%
24	Prolow-density lipoprotein receptor-related protein 1	LRP1_HUMAN	505 kDa	0.02	0.02	0.02	0.02	0.01	0.02	104	47	68	120	74	110	25	10	14	24	16	17	7.28%	2.75%	3.85%	5.92%	4.09%	4.40%
25	Myosin-9	MYH9_HUMAN	227 kDa	0.06	0.00	0.02	0.12	0.02	0.08	135	0	26	324	49	172	36	0	9	61	17	37	21.00%	0	6.94%	28.90%	12.30%	22.60%
26	Inter-alpha-trypsin inhibitor heavy chain H3	ITIH3_HUMAN	100 kDa	0.02	0.14	0.10	0.03	0.10	0.07	16	79	59	33	104	71	5	8	8	7	10	11	2.92%	6.63%	6.63%	3.93%	7.53%	8.88%
27	Vimentin	VIME_HUMAN	54 kDa	0.08	0.07	0.07	0.23	0.31	0.21	40	20	21	149	162	111	10	3	5	27	23	21	24.20%	7.30%	11.40%	50.00%	44.80%	41.40%
28	Alpha-fetoprotein	J3KMX3_HUMAN	70 kDa	0.06	0.28	0.21	0.04	0.16	0.15	39	110	87	33	110	106	3	6	6	3	6	5	6.27%	9.16%	9.16%	6.27%	9.16%	7.88%
29	Hemoglobin subunit alpha	HBA_HUMAN	15 kDa	0.32	0.82	0.96	0.43	0.50	0.59	50	74	90	84	81	94	7	7	7	7	7	7	34.50%	34.50%	34.50%	34.50%	34.50%	34.50%
30	Heat shock protein HSP 90-alpha	HS90A_HUMAN	85 kDa	0.03	0.11	0.10	0.11	0.14	0.14	68	85	90	208	217	215	6	7	9	17	17	16	20.10%	20.50%	23.60%	41.00%	42.60%	37.30%
31	Tubulin alpha-1C chain	F5H5D3_HUMAN	58 kDa	0.16	0.14	0.20	0.16	0.16	0.14	89	46	68	116	93	83	11	7	6	12	9	11	24.50%	17.50%	15.20%	24.50%	20.60%	22.50%
32	Apolipoprotein B-100	APOB_HUMAN	516 kDa	0.02	0.02	0.02	0.01	0.01	0.02	89	46	56	58	58	89	13	8	11	12	11	11	2.30%	2.04%	2.50%	2.30%	2.30%	2.30%
33	Alpha-actinin-1	ACTN1_HUMAN	103 kDa	0.12	0.04	0.05	0.16	0.07	0.10	168	51	52	262	105	154	21	3	6	34	18	23	34.10%	10.10%	11.20%	45.40%	34.50%	34.20%
34	Glyceraldehyde-3-phosphate dehydrogenase	G3P_HUMAN	36 kDa	0.18	0.20	0.22	0.32	0.29	0.27	65	43	49	144	110	103	10	6	7	17	14	13	38.20%	34.60%	41.20%	52.80%	46.60%	46.30%
35	Thrombospondin-1	TSP1_HUMAN	129 kDa	0.02	0.10	0.11	0.02	0.08	0.08	24	75	86	30	105	101	7	11	14	6	17	14	7.78%	12.20%	14.80%	7.09%	16.70%	15.50%
36	Putative elongation factor 1-alpha-like 3	EF1A3_HUMAN	50 kDa	0.13	0.15	0.22	0.17	0.20	0.18	63	44	68	108	103	93	10	6	7	14	11	13	25.10%	9.74%	12.30%	27.10%	19.90%	27.10%
37	Chitinase-3-like protein 1	CH3L1_HUMAN	43 kDa	0.12	0.43	0.28	0.10	0.23	0.11	52	104	72	51	98	49	11	12	12	9	14	9	32.10%	29.20%	29.20%	29.80%	37.30%	29.20%
38	Vinculin	VINC_HUMAN	124 kDa	0.04	0.11	0.09	0.08	0.08	0.09	48	81	65	125	109	113	12	12	12	22	18	20	14.40%	13.10%	14.10%	24.20%	17.70%	23.20%
39	Moesin	MOES_HUMAN	68 kDa	0.04	0.07	0.07	0.13	0.15	0.17	27	27	26	103	101	108	8	3	5	19	15	14	18.00%	5.20%	10.90%	26.90%	19.60%	19.60%
40	Transforming growth factor-beta-induced protein ig-h3	BGH3_HUMAN	75 kDa	0.15	0.13	0.11	0.06	0.07	0.06	108	57	48	53	51	46	12	12	11	10	11	8	20.60%	18.20%	18.20%	18.20%	19.20%	9.81%
41	Pigment epithelium-derived factor	PEDF_HUMAN	46 kDa	0.06	0.27	0.29	0.09	0.18	0.14	28	71	79	50	86	68	4	6	6	5	5	6	9.33%	12.20%	12.20%	12.20%	12.20%	12.20%
42	Cathepsin D	CATD_HUMAN	45 kDa	0.24	0.21	0.15	0.17	0.17	0.19	109	55	41	97	79	88	13	10	9	14	11	13	24.50%	24.30%	24.30%	24.50%	24.30%	24.50%
43	Complement C5	CO5_HUMAN	188 kDa	0.05	0.01	0.02	0.02	0.01	0.01	84	12	18	53	15	18	7	2	3	8	4	2	3.82%	2.33%	2.92%	4.89%	2.98%	2.21%
44	Prelamin-A/C	LMNA_HUMAN	74 kDa	0.07	0.00	0.02	0.14	0.04	0.09	53	0	10	126	32	69	11	0	2	23	7	16	18.70%	0	3.77%	33.30%	12.00%	28.60%
45	Ras GTPase-activating-like protein IQGAP1	IQGA1_HUMAN	189 kDa	0.04	0.00	0.00	0.04	0.02	0.03	64	3	4	90	35	53	16	3	2	27	13	15	15.40%	3.92%	2.84%	19.60%	13.20%	13.80%
46	Elongation factor 2	EF2_HUMAN	95 kDa	0.05	0.05	0.09	0.08	0.07	0.09	44	26	53	89	71	83	7	4	7	13	10	10	11.30%	7.11%	12.40%	17.10%	11.80%	13.40%
47	Antithrombin-III	ANT3_HUMAN	53 kDa	0.15	0.08	0.08	0.06	0.05	0.07	78	25	23	41	28	36	4	6	4	5	5	6	6.47%	9.05%	6.47%	8.84%	11.40%	11.40%
48	78 kDa glucose-regulated protein	GRP78_HUMAN	72 kDa	0.05	0.03	0.06	0.14	0.09	0.11	69	28	55	150	91	109	5	3	6	15	12	9	15.60%	8.87%	16.70%	30.90%	28.30%	21.70%
49	Inter-alpha-trypsin inhibitor heavy chain H1	ITIH1_HUMAN	101 kDa	0.03	0.12	0.10	0.05	0.08	0.06	30	69	63	63	86	62	6	7	8	8	8	7	3.95%	3.95%	3.95%	3.95%	3.95%	3.95%
50	Contactin-1	CNTN1_HUMAN	113 kDa	0.01	0.09	0.08	0.05	0.05	0.07	11	57	54	71	53	77	5	8	9	10	11	13	7.66%	8.84%	9.63%	12.30%	13.00%	15.30%
51	Collagen alpha-1(VI) chain	CO6A1_HUMAN	109 kDa	0.00	0.11	0.10	0.00	0.06	0.05	0	70	65	0	75	60	0	5	6	0	6	5	0	6.91%	7.30%	0	8.27%	6.52%
52	Actin-related protein 3	ARP3_HUMAN	47 kDa	0.15	0.00	0.04	0.12	0.05	0.08	68	0	11	70	25	39	12	0	3	13	6	9	29.40%	0	7.89%	31.60%	15.80%	25.10%
53	Actin, alpha cardiac muscle 1	ACTC_HUMAN	42 kDa	0.08	0.16	0.00	0.07	0.00	0.09	143	125	0	177	0	163	6	5	0	5	0	4	32.60%	30.00%	0	30.00%	0	33.20%
54	Actin-related protein 2	ARP2_HUMAN	45 kDa	0.20	0.05	0.09	0.12	0.09	0.10	87	12	23	67	38	45	10	2	6	9	6	8	29.70%	11.90%	18.00%	29.70%	21.80%	26.10%

55	Fibulin-1	FBLN1_HUMAN	77 kDa	0.02	0.05	0.06	0.03	0.04	0.05	19	21	28	28	28	40	4	3	3	4	4	5	7.25%	3.27%	3.27%	6.40%	4.41%	5.83%
56	Protocadherin Fat 4	FAT4_HUMAN	543 kDa	0.00	0.00	0.00	0.00	0.00	0.00	0	0	0	6	0	13	0	0	0	3	0	5	0	0	0	1.10%	0	1.87%
57	Adenosylhomocysteinase	SAHH_HUMAN	48 kDa	0.06	0.08	0.13	0.10	0.12	0.11	30	22	36	57	59	54	5	4	7	7	8	5	13.40%	11.10%	19.00%	19.40%	18.80%	14.60%
58	Coronin-1A	COR1A_HUMAN	51 kDa	0.09	0.10	0.10	0.11	0.12	0.09	47	30	30	67	62	49	4	3	3	6	3	3	13.70%	11.90%	11.90%	17.80%	11.90%	11.90%
59	Fructose-bisphosphate aldolase A	ALDOA_HUMAN (+1)	39 kDa	0.03	0.13	0.07	0.06	0.16	0.11	11	30	18	30	65	43	4	6	7	7	7	7	11.00%	22.50%	23.90%	15.90%	24.20%	21.70%
60	Keratin, type II cytoskeletal 5	K2C5_HUMAN	62 kDa	0.10	0.07	0.00	0.04	0.02	0.06	108	53	0	57	40	60	11	5	0	6	4	5	22.20%	11.40%	0	13.10%	12.90%	13.10%
61	Ubiquitin-like modifier-activating enzyme 1	UBA1_HUMAN	118 kDa	0.02	0.00	0.00	0.07	0.04	0.06	18	0	3	103	47	69	5	0	2	16	9	15	6.52%	0	3.40%	22.70%	12.10%	19.30%
62	Matrix metalloproteinase-9	MMP9_HUMAN	78 kDa	0.01	0.16	0.06	0.02	0.08	0.06	10	70	29	22	62	45	2	10	7	4	11	9	3.68%	19.90%	12.40%	7.50%	18.00%	17.00%
63	Plastin-2	PLSL_HUMAN	70 kDa	0.02	0.08	0.03	0.05	0.15	0.05	14	30	12	46	105	35	3	7	3	13	17	10	7.18%	15.20%	7.18%	25.50%	31.70%	24.10%
64	Transitional endoplasmic reticulum ATPase	TERA_HUMAN	89 kDa	0.07	0.00	0.02	0.12	0.00	0.06	61	0	10	130	3	54	17	0	3	23	2	10	23.10%	0	5.33%	31.30%	3.72%	16.70%
65	Fatty acid synthase	FAS_HUMAN	273 kDa	0.03	0.00	0.00	0.02	0.01	0.02	75	0	0	70	17	63	17	0	0	15	7	13	10.40%	0	0	9.80%	4.94%	8.20%
66	Catalase	CATA_HUMAN	60 kDa	0.14	0.04	0.08	0.09	0.05	0.08	82	15	28	65	28	50	8	3	4	9	4	7	21.30%	7.59%	10.40%	24.30%	13.30%	21.30%
67	Proteasome subunit alpha type-7	PSA7_HUMAN	28 kDa	0.18	0.10	0.14	0.13	0.09	0.09	48	16	23	43	26	25	8	4	4	7	6	4	44.00%	25.00%	25.00%	36.30%	36.30%	25.00%
68	Clathrin heavy chain	A0A087WVQ6_HUMAI	192 kDa	0.05	0.00	0.00	0.03	0.01	0.03	97	0	0	68	10	48	25	0	0	17	5	13	20.10%	0	0	14.80%	3.28%	11.10%
69	Ceruloplasmin	CERU_HUMAN	122 kDa	0.03	0.04	0.03	0.05	0.02	0.02	31	28	22	68	26	24	7	5	4	9	5	5	6.85%	5.07%	4.69%	7.79%	5.07%	5.73%
70	Lumican	LUM_HUMAN	38 kDa	0.00	0.00	0.27	0.00	0.00	0.13	0	0	61	0	0	50	0	0	3	0	0	3	0	0	8.28%	0	0	8.28%
71	Vitronectin	VTNC_HUMAN	54 kDa	0.07	0.12	0.14	0.07	0.06	0.07	38	37	45	48	31	40	3	3	4	3	4	4	5.02%	5.02%	6.69%	5.02%	6.69%	6.69%
72	Pregnancy zone protein	PZP_HUMAN	164 kDa	0.01	0.02	0.02	0.02	0.00	0.00	145	134	133	189	0	0	2	2	2	2	0	0	4.72%	3.85%	3.85%	4.72%	0	0
73	Lactotransferrin	TRFL_HUMAN	78 kDa	0.03	0.05	0.05	0.02	0.04	0.03	21	21	25	20	36	27	2	2	3	2	3	2	2.54%	2.54%	4.37%	2.54%	4.37%	2.54%
74	T-complex protein 1 subunit zeta	TCPZ_HUMAN	58 kDa	0.08	0.00	0.02	0.09	0.08	0.09	48	0	6	66	48	55	11	0	4	13	8	9	24.90%	0	9.04%	27.30%	20.20%	22.60%
75	14-3-3 protein zeta/delta	1433Z_HUMAN	28 kDa	0.17	0.25	0.32	0.13	0.18	0.16	44	39	52	45	51	44	6	5	6	6	6	6	24.90%	24.90%	24.90%	24.90%	24.90%	24.90%
76	T-complex protein 1 subunit beta	TCPB_HUMAN	57 kDa	0.10	0.00	0.02	0.08	0.06	0.07	57	0	6	55	36	40	12	0	3	9	6	6	31.40%	0	7.48%	22.20%	13.30%	14.60%
77	ITIH4 protein	B7ZKJ8_HUMAN (+1)	104 kDa	0.00	0.00	0.00	0.01	0.01	0.01	0	0	0	8	15	12	0	0	0	3	4	3	0	0	0	2.46%	2.46%	2.46%
78	Phosphoglycerate kinase 1	PGK1_HUMAN	45 kDa	0.07	0.05	0.07	0.07	0.07	0.08	33	13	19	38	31	36	6	4	4	6	6	7	20.90%	15.30%	15.30%	20.90%	21.60%	23.30%
79	Actin-related protein 2/3 complex subunit 2	ARPC2_HUMAN	34 kDa	0.17	0.03	0.04	0.10	0.06	0.05	56	6	7	41	21	17	8	2	2	9	4	5	30.70%	16.00%	16.00%	31.70%	22.30%	19.00%
80	Collagen alpha-1(I) chain	CO1A1_HUMAN	139 kDa	0.01	0.03	0.04	0.01	0.03	0.02	9	25	34	15	49	33	4	4	5	3	5	4	2.39%	2.39%	3.28%	2.39%	3.28%	2.39%
81	Transketolase	TKT_HUMAN	68 kDa	0.00	0.02	0.03	0.06	0.10	0.08	0	6	12	51	74	57	0	3	4	8	8	7	0	8.03%	10.80%	13.00%	13.00%	13.00%
82	Eukaryotic initiation factor 4A-I	IF4A1_HUMAN	46 kDa	0.06	0.00	0.06	0.14	0.09	0.12	25	0	15	76	42	53	4	0	5	12	9	9	13.30%	0	14.30%	33.50%	26.60%	25.40%
83	T-complex protein 1 subunit epsilon	TCPE_HUMAN	60 kDa	0.08	0.00	0.03	0.05	0.07	0.06	46	0	9	38	41	36	6	0	3	6	5	5	15.30%	0	7.39%	14.20%	12.40%	12.00%
84	Nucleolin	NUCL_HUMAN	77 kDa	0.02	0.00	0.00	0.05	0.07	0.07	17	0	0	52	53	56	4	0	0	9	7	8	6.76%	0	0	15.90%	12.70%	13.10%
85	Rab GDP dissociation inhibitor beta	GDIB_HUMAN	51 kDa	0.02	0.11	0.14	0.07	0.08	0.09	12	30	40	41	38	47	3	5	5	6	6	6	11.00%	15.30%	15.30%	18.00%	19.80%	15.50%
86	Tubulin beta-4B chain	TBB4B_HUMAN	50 kDa	0.08	0.04	0.10	0.10	0.09	0.09	141	71	117	206	188	179	6	4	6	7	6	8	56.90%	34.60%	58.20%	67.40%	63.40%	60.00%
87	Proteasome subunit alpha type	A0A024RA52_HUMAN	26 kDa	0.30	0.09	0.15	0.21	0.11	0.06	77	13	23	67	28	16	8	2	3	7	5	3	37.20%	17.10%	17.10%	32.90%	17.50%	17.10%
88	Histone H2A type 1-B/E	H2A1B_HUMAN (+2)	14 kDa	0.31	0.00	0.30	0.40	0.12	0.37	44	0	26	71	18	54	4	0	4	5	2	5	43.80%	0	43.80%	43.80%	36.90%	43.80%
89	Carbonic anhydrase 2	CAH2_HUMAN	29 kDa	0.05	0.22	0.12	0.08	0.19	0.09	13	36	20	28	57	25	3	6	3	4	9	3	16.90%	31.20%	16.90%	16.90%	36.20%	16.90%
90	Collagen alpha-2(I) chain	A0A087WTA8_HUMAN	129 kDa	0.00	0.02	0.02	0.00	0.02	0.01	0	20	18	0	28	18	0	2	2	0	5	3	0	2.20%	2.20%	0	4.25%	2.86%
91	Nucleophosmin	NPM_HUMAN	33 kDa	0.15	0.00	0.00	0.11	0.12	0.07	47	0	0	45	40	23	8	0	0	8	6	5	24.10%	0	0	24.50%	19.70%	10.20%
92	Heat shock 70 kDa protein 1B	A0A0G2JIW1_HUMAN	70 kDa	0.04	0.00	0.02	0.05	0.02	0.04	51	0	24	65	33	43	3	0	2	12	5	5	11.10%	0	8.57%	23.80%	14.00%	12.50%
93	Fermitin family homolog 3	URP2_HUMAN	76 kDa	0.01	0.00	0.00	0.06	0.03	0.05	5	0	0	53	25	34	4	0	0	8	6	8	8.70%	0	0	23.20%	14.70%	22.20%
94	Proteasome subunit alpha type-1	PSA1_HUMAN	30 kDa	0.22	0.00	0.00	0.13	0.02	0.05	62	0	0	46	5	14	12	0	0	9	3	5	39.90%	0	0	36.50%	18.60%	25.10%
95	Complement component C9	CO9_HUMAN	63 kDa	0.05	0.09	0.08	0.04	0.05	0.05	28	32	30	34	31	33	2	2	2	2	2	3	3.76%	3.76%	3.76%	3.76%	3.76%	4.11%
96	UTP--glucose-1-phosphate uridylyltransferase	UGPA_HUMAN	57 kDa	0.06	0.01	0.00	0.04	0.00	0.03	31	3	0	26	0	15	7	2	0	6	0	4	17.50%	6.69%	0	20.30%	0	12.60%
97	DNA-dependent protein kinase catalytic subunit	PRKDC_HUMAN	469 kDa	0.00	0.00	0.00	0.00	0.00	0.00	2	0	0	0	0	0	2	0	0	0	0	0	1.07%	0	0	0	0	0
98	6-phosphogluconate dehydrogenase, decarboxylating	6PGD_HUMAN	53 kDa	0.01	0.00	0.05	0.07	0.08	0.08	7	0	16	46	44	41	4	0	5	6	6	6	9.11%	0	13.30%	16.80%	16.80%	16.80%
99	Dihydropyrimidinase-related protein 2	DPYL2_HUMAN	62 kDa	0.04	0.00	0.01	0.08	0.01	0.02	24	0	2	60	6	15	5	0	2	12	2	5	17.80%	0	8.22%	29.20%	8.22%	17.80%
100	Heterogeneous nuclear ribonucleoprotein K	HNRPK_HUMAN	51 kDa	0.01	0.00	0.00	0.09	0.07	0.07	4	0	0	54	35	38	2	0	0	5	5	5	6.05%	0	0	17.70%	17.30%	14.50%
101	Argininosuccinate synthase	ASSY_HUMAN	47 kDa	0.00	0.07	0.14	0.02	0.09	0.06	0	19	39	10	42	26	0	3	5	3	6	6	0	10.40%	12.40%	10.70%	15.50%	12.40%
102	Epididymis luminal protein 189	Q5HYB6_HUMAN	27 kDa	0.06	0.09	0.10	0.05	0.16	0.05	14	13	16	15	41	12	2	2	4	2	7	3	6.47%	6.47%	14.20%	6.47%	19.00%	11.20%
103	ATP-citrate synthase	ACLY_HUMAN	121 kDa	0.03	0.00	0.00	0.03	0.00	0.01	35	0	0	38	3	9	12	0	0	7	2	3	13.50%	0	0	9.17%	3.36%	3.54%
104	Fructose-bisphosphate aldolase	A8MVZ9_HUMAN	36 kDa	0.04	0.11	0.11	0.04	0.09	0.08	13	23	24	20	36	29	3	3	4	3	4	3	7.74%	7.74%	11.90%	7.74%	11.90%	7.74%
105	Filamin-A	Q5HY54_HUMAN	277 kDa	0.00	0.00	0.00	0.00	0.00	0.02	5	0	8	10	12	45	3	0	6	4	4	10	1.96%	0	4.41%	2.61%	2.42%	6.48%
106	GTP-binding nuclear protein Ran (Fragment)	J3KQE5_HUMAN	27 kDa	0.06	0.04	0.08	0.06	0.04	0.05	15	6	13	19	10	14	2	2	2	3	2	2	10.70%	10.70%	10.70%	15.00%	10.70%	10.70%
107	Elongation factor 1-gamma	EF1G_HUMAN	50 kDa	0.04	0.02	0.02	0.04	0.02	0.05	20	5	6	23	12	25	5	2	3	5	4	5	10.50%	5.03%	8.24%	10.50%	10.50%	10.50%
108	Tenascin	J3QSU6_HUMAN (+1)	221 kDa	0.00	0.00	0.00	0.00	0.00	0.01	0	5	0	0	0	24	0	2	0	0	0	5	0	2.13%	0	0	0	3.02%

109	Cation-independent mannose-6-phosphate receptor	MPRI_HUMAN	274 kDa	0.00	0.01	0.01	0.00	0.00	0.01	8	9	9	16	12	16	2	2	2	2	3	2	0.72%	0.72%	0.72%	0.72%	0.76%	0.72%
110	X-ray repair cross-complementing protein 6	B1AHC9_HUMAN	64 kDa	0.01	0.00	0.00	0.07	0.03	0.08	8	0	0	54	19	51	3	0	0	9	6	7	8.41%	0	0	17.20%	13.10%	15.40%
111	Valine--tRNA ligase	SYVC_HUMAN	140 kDa	0.01	0.00	0.00	0.02	0.00	0.01	8	0	0	35	3	12	3	0	0	10	3	4	4.03%	0	0	10.70%	3.56%	6.17%
112	Nucleosome assembly protein 1-like 1	NP1L1_HUMAN	45 kDa	0.06	0.02	0.07	0.07	0.05	0.04	26	5	18	39	20	18	3	2	2	9	2	4	14.60%	7.16%	7.16%	29.90%	7.16%	13.00%
113	Lysosomal alpha-mannosidase	MA2B1_HUMAN	114 kDa	0.02	0.00	0.01	0.02	0.00	0.03	24	0	6	29	5	30	7	0	5	8	4	8	11.80%	0	6.82%	11.80%	5.64%	11.00%
114	Proteasome subunit beta type-1	PSB1_HUMAN	26 kDa	0.16	0.04	0.08	0.14	0.11	0.09	41	6	13	45	31	23	8	2	3	6	4	4	34.90%	14.10%	14.10%	23.70%	14.10%	14.10%
115	Annexin A1	ANXA1_HUMAN	39 kDa	0.07	0.15	0.02	0.06	0.12	0.03	28	32	4	28	47	10	5	6	3	5	9	4	17.30%	24.30%	13.00%	19.70%	29.80%	17.30%
116	Lipoprotein lipase	LIPL_HUMAN	53 kDa	0.05	0.04	0.12	0.00	0.00	0.03	25	12	38	0	0	15	7	3	8	0	0	3	19.20%	10.30%	19.20%	0	0	10.30%
117	Thrombospondin-4	E7ES19_HUMAN (+1)	96 kDa	0.00	0.00	0.01	0.01	0.01	0.01	0	0	5	13	19	17	0	0	2	2	5	3	0	0	3.56%	3.68%	6.67%	5.17%
118	T-complex protein 1 subunit gamma	TCPG_HUMAN	61 kDa	0.03	0.00	0.00	0.03	0.00	0.02	17	0	0	24	0	14	4	0	0	5	0	3	10.80%	0	0	15.00%	0	8.81%
119	F-actin-capping protein subunit alpha-1	CAZA1_HUMAN	33 kDa	0.09	0.05	0.08	0.08	0.09	0.07	29	9	15	31	28	24	3	2	3	4	5	5	17.10%	11.50%	17.10%	20.60%	29.70%	29.70%
120	Betaine--homocysteine S-methyltransferase 1	BHMT1_HUMAN	45 kDa	0.04	0.08	0.11	0.03	0.04	0.04	16	21	29	14	19	20	2	4	3	2	2	2	6.40%	13.80%	8.62%	6.16%	6.40%	6.40%
121	L-lactate dehydrogenase B chain	LDHB_HUMAN	37 kDa	0.05	0.10	0.06	0.05	0.07	0.05	17	22	13	23	26	17	4	5	4	5	7	4	14.70%	16.80%	12.60%	19.50%	22.50%	16.20%
122	Palmitoyl-protein thioesterase 1	PPT1_HUMAN	34 kDa	0.14	0.08	0.10	0.06	0.05	0.04	45	16	20	25	19	15	5	4	5	4	4	3	21.90%	21.90%	21.90%	21.90%	21.90%	21.90%
123	Apolipoprotein A-IV	APOA4_HUMAN	45 kDa	0.05	0.07	0.08	0.06	0.03	0.03	22	18	20	31	15	15	2	2	2	3	2	2	5.81%	5.81%	5.81%	5.81%	5.81%	5.81%
124	Uncharacterized protein	B4E1Z4_HUMAN	141 kDa	0.00	0.00	0.00	0.00	0.00	0.00	0	0	0	7	0	0	0	0	0	2	0	0	0	0	0	1.66%	0	0
125	Hsp90 co-chaperone Cdc37	CDC37_HUMAN	44 kDa	0.00	0.00	0.02	0.02	0.00	0.03	0	0	6	9	0	11	0	0	3	3	0	3	0	0	7.94%	7.94%	0	7.94%
126	Actin-related protein 2/3 complex subunit 4	ARPC4_HUMAN (+1)	20 kDa	0.20	0.00	0.00	0.20	0.00	0.00	37	0	0	45	0	0	5	0	0	5	0	0	41.70%	0	0	41.70%	0	0
127	T-complex protein 1 subunit alpha	TCPA_HUMAN	60 kDa	0.03	0.00	0.00	0.05	0.02	0.03	18	0	0	35	12	17	5	0	0	7	3	2	19.40%	0	0	15.30%	5.76%	5.76%
128	T-complex protein 1 subunit theta	TCPQ_HUMAN	60 kDa	0.06	0.00	0.00	0.04	0.03	0.03	36	0	0	27	16	20	9	0	0	5	3	5	19.70%	0	0	11.30%	7.12%	11.10%
129	14-3-3 protein gamma	1433G_HUMAN	28 kDa	0.07	0.10	0.13	0.07	0.13	0.10	24	23	32	36	49	36	3	4	3	4	4	3	17.40%	17.40%	17.40%	21.10%	21.10%	17.40%
130	Heterogeneous nuclear ribonucleoprotein U	HNRPU_HUMAN	91 kDa	0.00	0.00	0.00	0.00	0.00	0.03	0	0	0	0	0	29	0	0	0	0	0	2	0	0	0	0	0	4.12%
131	Nucleobindin-1	NUCB1_HUMAN	54 kDa	0.00	0.07	0.04	0.00	0.04	0.03	0	21	13	0	19	13	0	4	3	0	4	3	0	13.70%	11.70%	0	13.70%	11.70%
132	BTB/POZ domain-containing protein KCTD12	KCD12_HUMAN	36 kDa	0.03	0.00	0.03	0.05	0.04	0.04	11	0	7	22	16	13	4	0	2	5	4	3	18.80%	0	7.38%	21.80%	18.50%	12.00%
133	Basement membrane-specific heparan sulfate proteoglycan core protein	PGBM_HUMAN	469 kDa	0.00	0.00	0.01	0.00	0.00	0.01	5	0	15	0	20	33	2	0	4	0	5	6	0.73%	0	1.43%	0	1.43%	1.96%
134	Bifunctional glutamate/proline--tRNA ligase	SYEP_HUMAN	171 kDa	0.00	0.00	0.00	0.00	0.00	0.01	5	0	0	10	6	25	4	0	0	4	3	6	4.56%	0	0	4.76%	2.58%	6.94%
135	Cytosol aminopeptidase	AMPL_HUMAN	56 kDa	0.00	0.00	0.00	0.04	0.03	0.06	0	0	0	25	18	34	0	0	0	6	3	7	0	0	0	14.80%	9.06%	17.90%
136	Actin-related protein 2/3 complex subunit 1B	ARC1B_HUMAN	41 kDa	0.05	0.00	0.00	0.00	0.00	0.03	20	0	0	0	0	13	3	0	0	0	0	3	8.33%	0	0	0	0	9.95%
137	60 kDa heat shock protein, mitochondrial	CH60_HUMAN	61 kDa	0.00	0.00	0.00	0.02	0.01	0.01	0	0	0	13	9	5	0	0	0	5	2	3	0	0	0	14.30%	8.55%	10.60%
138	Lamin-B1	LMNB1_HUMAN	66 kDa	0.01	0.00	0.00	0.03	0.02	0.02	6	0	0	28	10	10	2	0	0	8	3	2	9.22%	0	0	19.50%	8.70%	6.48%
139	Cytoplasmic dynein 1 heavy chain 1	DYHC1_HUMAN	532 kDa	0.00	0.00	0.00	0.00	0.00	0.00	19	0	0	16	0	14	10	0	0	6	0	7	3.53%	0	0	2.56%	0	2.28%
140	26S proteasome non-ATPase regulatory subunit 2	PSMD2_HUMAN	100 kDa	0.01	0.00	0.00	0.03	0.01	0.04	13	0	0	41	14	42	5	0	0	6	4	7	7.93%	0	0	8.15%	6.61%	8.15%
141	Tetranectin	E9PHK0_HUMAN (+1)	18 kDa	0.00	0.25	0.24	0.00	0.13	0.00	0	25	25	0	24	0	0	3	3	0	2	0	0	21.90%	21.90%	0	21.90%	0
142	Keratin, type I cytoskeletal 14	K1C14_HUMAN	52 kDa	0.08	0.05	0.00	0.04	0.02	0.05	90	49	0	42	29	38	10	2	0	2	2	3	37.90%	12.30%	0	7.84%	7.84%	9.96%
143	T-complex protein 1 subunit delta	TCPD_HUMAN	58 kDa	0.03	0.00	0.00	0.03	0.02	0.03	17	0	0	22	11	18	2	0	0	6	2	3	5.38%	0	0	15.00%	4.45%	6.86%
144	L-amino-acid oxidase	OXLA_HUMAN	63 kDa	0.00	0.00	0.00	0.02	0.02	0.01	0	0	0	17	12	8	0	0	0	6	6	2	0	0	0	12.70%	12.70%	6.70%
145	Heat shock 70 kDa protein 4	HSP74_HUMAN	94 kDa	0.00	0.00	0.00	0.01	0.01	0.01	0	0	0	12	13	13	0	0	0	2	5	3	0	0	0	4.17%	8.69%	5.48%
146	Proteoglycan 4	PRG4_HUMAN	151 kDa	0.01	0.00	0.00	0.01	0.00	0.00	17	0	0	22	0	0	3	0	0	3	0	0	3.13%	0	0	3.13%	0	0
147	Cathepsin S	CATS_HUMAN	37 kDa	0.03	0.03	0.04	0.03	0.06	0.04	11	6	8	14	22	15	2	2	2	3	3	2	5.74%	6.34%	6.34%	8.76%	8.76%	6.34%
148	Cartilage oligomeric matrix protein	COMP_HUMAN (+1)	83 kDa	0.00	0.03	0.03	0.00	0.03	0.02	0	22	18	0	31	23	0	3	3	0	3	2	0	6.21%	6.21%	0	7.93%	4.49%
149	Protein disulfide-isomerase A3	PDIA3_HUMAN	57 kDa	0.00	0.00	0.00	0.05	0.04	0.03	0	0	0	34	24	17	0	0	0	8	5	4	0	0	0	18.00%	13.10%	10.30%
150	Prosaposin	SAP_HUMAN	58 kDa	0.05	0.00	0.00	0.01	0.00	0.02	30	0	0	7	0	10	6	0	0	3	0	3	13.70%	0	0	10.70%	0	10.70%
151	Endoplasmin	ENPL_HUMAN	92 kDa	0.00	0.00	0.00	0.01	0.03	0.02	0	0	0	30	45	37	0	0	0	3	7	3	0	0	0	5.98%	14.30%	7.85%
152	Serine/threonine-protein phosphatase 2A 65 kDa regulatory subunit A alpha isoform	2AAA_HUMAN	65 kDa	0.00	0.00	0.00	0.01	0.01	0.01	3	0	0	7	7	9	2	0	0	3	3	4	4.75%	0	0	6.45%	8.15%	9.85%

153	Carboxypeptidase N catalytic chain	CBPN_HUMAN	52 kDa	0.00	0.05	0.05	0.00	0.02	0.02	0	15	14	0	12	12	0	3	2	0	3	2	0	6.33%	6.33%	0	6.33%	6.33%
154	Bifunctional purine biosynthesis protein PURH	PUR9_HUMAN	65 kDa	0.00	0.00	0.00	0.04	0.01	0.03	0	0	0	32	8	22	0	0	0	10	3	5	0	0	0	24.50%	9.29%	15.00%
155	Receptor-type tyrosine-protein phosphatase F	PTPRF_HUMAN	213 kDa	0.00	0.00	0.00	0.00	0.00	0.00	3	0	0	10	0	5	2	0	0	3	0	3	1.57%	0	0	2.15%	0	2.15%
156	C-type lectin domain family 11 member A	A0A0A6YY95_HUMAN	36 kDa	0.05	0.02	0.10	0.00	0.00	0.00	19	5	22	0	0	0	5	2	4	0	0	0	19.80%	7.12%	14.60%	0	0	0
157	T-complex protein 1 subunit eta	TCPH_HUMAN	59 kDa	0.07	0.00	0.00	0.03	0.00	0.04	39	0	0	24	0	25	5	0	0	3	0	4	11.40%	0	0	8.47%	0	9.94%
158	Proteasome subunit beta type-3 (Fragment)	A0A087WUL2_HUMAN	16 kDa	0.21	0.00	0.00	0.14	0.00	0.00	33	0	0	27	0	0	4	0	0	3	0	0	33.10%	0	0	33.10%	0	0
159	Fructose-bisphosphate aldolase B	ALDOB_HUMAN	39 kDa	0.00	0.12	0.09	0.00	0.06	0.05	0	28	22	0	24	20	0	5	4	0	4	3	0	11.50%	8.79%	0	8.79%	8.79%
160	Ras-related protein Rap-1A	RAP1A_HUMAN	21 kDa	0.07	0.11	0.00	0.07	0.00	0.00	14	13	0	17	0	0	2	2	0	2	0	0	12.50%	12.50%	0	12.50%	0	0
161	14-3-3 protein epsilon	1433E_HUMAN	29 kDa	0.00	0.00	0.00	0.00	0.08	0.00	0	0	0	0	36	0	0	0	0	0	3	0	0	0	0	0	16.10%	0
162	Heat shock protein beta-1	HSPB1_HUMAN	23 kDa	0.06	0.00	0.16	0.07	0.08	0.08	13	0	22	20	18	19	2	0	2	4	3	2	13.20%	0	13.20%	36.10%	21.00%	13.20%
163	Glycogen phosphorylase, liver form	PYGL_HUMAN	97 kDa	0.00	0.02	0.01	0.01	0.01	0.02	0	9	7	9	10	19	0	2	2	2	3	3	0	3.07%	3.07%	3.07%	4.49%	4.49%
164	60S acidic ribosomal protein P0	RLA0_HUMAN	34 kDa	0.00	0.00	0.00	0.06	0.07	0.06	0	0	0	26	26	21	0	0	0	5	7	5	0	0	0	23.30%	32.20%	23.70%
165	Tryptophan--tRNA ligase, cytoplasmic	SYWC_HUMAN	53 kDa	0.00	0.00	0.00	0.03	0.04	0.02	0	0	0	20	23	10	0	0	0	4	3	3	0	0	0	10.80%	10.80%	10.80%
166	Neogenin	NEO1_HUMAN	160 kDa	0.00	0.01	0.01	0.01	0.00	0.02	0	12	10	18	6	25	0	6	4	5	3	5	0	7.05%	5.13%	4.72%	3.97%	5.20%
167	Keratin, type I cytoskeletal 16	K1C16_HUMAN	51 kDa	0.10	0.01	0.00	0.00	0.00	0.00	75	34	0	0	0	0	13	2	0	0	0	0	35.70%	6.34%	0	0	0	0
168	Spectrin alpha chain, non-erythrocytic 1	SPTN1_HUMAN	285 kDa	0.00	0.00	0.00	0.00	0.00	0.00	0	0	0	4	0	0	0	0	0	3	0	0	0	0	0	2.06%	0	0
169	Collagen alpha-1(II) chain	CO2A1_HUMAN	142 kDa	0.00	0.00	0.00	0.00	0.00	0.00	0	0	2	0	0	0	0	0	2	0	0	0	0	0	1.61%	0	0	0
170	Collagen alpha-1(XII) chain	COCA1_HUMAN (+1)	333 kDa	0.00	0.00	0.00	0.00	0.00	0.01	0	0	0	0	11	18	0	0	0	0	3	5	0	0	0	0	1.53%	2.51%
171	Importin subunit beta-1	IMB1_HUMAN	97 kDa	0.01	0.00	0.01	0.01	0.01	0.02	13	0	6	14	14	18	3	0	2	3	2	2	6.74%	0	3.08%	3.08%	3.08%	3.08%
172	Cullin-associated NEDD8-dissociated protein 1	CAND1_HUMAN	136 kDa	0.00	0.00	0.00	0.00	0.00	0.00	0	0	0	3	0	5	0	0	0	3	0	2	0	0	0	3.33%	0	3.33%
173	Protein disulfide-isomerase A6	PDIA6_HUMAN	48 kDa	0.01	0.00	0.00	0.06	0.05	0.03	4	0	0	34	24	17	3	0	0	5	3	3	14.30%	0	0	18.20%	14.30%	14.30%
174	4-trimethylaminobutyraldehyde dehydrogenase	AL9A1_HUMAN	54 kDa	0.00	0.02	0.04	0.00	0.03	0.02	0	7	14	0	16	11	0	2	2	0	3	3	0	4.45%	4.45%	0	6.88%	6.88%
175	Deoxynucleoside triphosphate triphosphohydrolase SAMHD1	SAMH1_HUMAN	72 kDa	0.00	0.00	0.00	0.01	0.00	0.00	0	0	0	10	0	0	0	0	0	2	0	0	0	0	0	3.99%	0	0
176	Chloride intracellular channel protein 1	CLIC1_HUMAN	27 kDa	0.04	0.00	0.08	0.04	0.00	0.05	11	0	12	14	0	14	2	0	2	4	0	2	12.40%	0	12.40%	28.60%	0	12.40%
177	Proteasome subunit beta type-4	PSB4_HUMAN	29 kDa	0.08	0.00	0.00	0.04	0.00	0.03	22	0	0	16	0	9	5	0	0	5	0	2	26.10%	0	0	26.10%	0	12.90%
178	Dermcidin	DCD_HUMAN	11 kDa	0.17	0.14	0.00	0.00	0.00	0.15	20	10	0	0	0	19	3	2	0	0	0	3	22.70%	20.00%	0	0	0	32.70%
179	Tenascin-X	E7EPZ9_HUMAN (+1)	458 kDa	0.00	0.00	0.00	0.00	0.00	0.00	0	0	0	0	0	3	0	0	0	0	0	2	0	0	0	0	0	0.52%
180	Proteasome subunit alpha type-6	PSA6_HUMAN	27 kDa	0.07	0.00	0.02	0.07	0.00	0.05	19	0	3	22	0	13	4	0	2	4	0	2	17.50%	0	12.60%	21.50%	0	12.60%
181	Proteasome subunit alpha type-3	PSA3_HUMAN	28 kDa	0.05	0.00	0.00	0.05	0.00	0.00	15	0	0	18	0	0	4	0	0	5	0	0	16.90%	0	0	16.90%	0	0
182	Collagen alpha-3(VI) chain	CO6A3_HUMAN	344 kDa	0.00	0.00	0.00	0.00	0.00	0.00	0	0	0	7	7	0	0	0	0	2	2	0	0	0	0	0.79%	0.79%	0
183	Sulfhydryl oxidase 1	QSOX1_HUMAN	83 kDa	0.00	0.02	0.00	0.02	0.00	0.00	0	10	0	17	0	0	0	2	0	2	0	0	0	4.28%	0	3.35%	0	0
184	Tubulin beta-6 chain	TBB6_HUMAN	50 kDa	0.03	0.00	0.00	0.05	0.01	0.02	47	0	0	84	53	45	6	0	0	8	3	3	41.90%	0	0	47.80%	31.20%	31.40%
185	40S ribosomal protein SA	A0A0C4DG17_HUMAN	33 kDa	0.06	0.00	0.00	0.04	0.06	0.05	21	0	0	17	21	17	3	0	0	3	4	3	12.70%	0	0	17.70%	20.30%	12.70%
186	X-ray repair cross-complementing protein 5	XRCC5_HUMAN	83 kDa	0.02	0.00	0.00	0.02	0.00	0.01	13	0	0	20	0	9	5	0	0	6	0	4	12.80%	0	0	7.65%	0	5.46%
187	Histone H4	H4_HUMAN	11 kDa	0.13	0.00	0.00	0.08	0.00	0.15	15	0	0	11	0	17	4	0	0	4	0	5	42.70%	0	0	42.70%	0	42.70%
188	Serpin H1	SERPH_HUMAN	46 kDa	0.00	0.00	0.00	0.00	0.01	0.00	0	0	0	0	6	0	0	0	0	0	2	0	0	0	0	0	5.74%	0
189	Laminin subunit beta-1	G3XAI2_HUMAN (+1)	200 kDa	0.00	0.00	0.00	0.00	0.00	0.00	0	0	0	0	0	10	0	0	0	0	0	2	0	0	0	0	0	1.44%
190	V-type proton ATPase catalytic subunit A	VATA_HUMAN	68 kDa	0.03	0.00	0.00	0.02	0.00	0.02	18	0	0	13	0	11	3	0	0	3	0	3	5.67%	0	0	5.35%	0	5.67%
191	Staphylococcal nuclease domain-containing protein 1	SND1_HUMAN	102 kDa	0.00	0.00	0.00	0.01	0.01	0.02	4	0	0	11	10	19	2	0	0	3	4	5	4.84%	0	0	6.59%	8.02%	9.34%
192	Synaptic vesicle membrane protein VAT-1 homolog	VAT1_HUMAN	42 kDa	0.00	0.00	0.00	0.03	0.00	0.00	0	0	0	17	0	0	0	0	0	3	0	0	0	0	0	10.40%	0	0
193	S-adenosylmethionine synthase isoform type-2	METK2_HUMAN	44 kDa	0.00	0.00	0.00	0.02	0.03	0.04	0	0	0	11	13	17	0	0	0	3	2	3	0	0	0	9.87%	7.59%	7.59%
194	Coronin-1B	COR1B_HUMAN	54 kDa	0.00	0.00	0.00	0.02	0.00	0.02	0	0	0	12	0	13	0	0	0	3	0	2	0	0	0	14.10%	0	9.00%
195	Heterogeneous nuclear ribonucleoprotein D0	HNRPD_HUMAN	38 kDa	0.00	0.00	0.00	0.00	0.00	0.03	0	0	0	0	0	11	0	0	0	0	0	3	0	0	0	0	0	11.30%
196	Glucose-6-phosphate isomerase (Fragment)	A0A0A0MTS2_HUMAN	65 kDa	0.00	0.00	0.00	0.00	0.03	0.00	0	0	0	0	22	0	0	0	0	0	5	0	0	0	0	0	8.20%	0

197	L-lactate dehydrogenase A chain	LDHA_HUMAN	37 kDa	0.00	0.01	0.02	0.02	0.07	0.04	0	2	4	9	25	14	0	2	2	3	5	4	0	8.73%	9.04%	9.34%	16.60%	16.60%
198	Multifunctional protein ADE2 (Fragment)	E9PBS1_HUMAN (+1)	46 kDa	0.01	0.00	0.00	0.03	0.00	0.00	4	0	0	19	0	0	4	0	0	2	0	0	13.30%	0	0	6.05%	0	0
199	Collagen alpha-1(III) chain	CO3A1_HUMAN	139 kDa	0.00	0.02	0.02	0.00	0.01	0.00	0	19	17	0	19	5	0	2	2	0	2	2	0	1.84%	1.84%	0	1.84%	1.84%
200	ATP-dependent RNA helicase A	DHX9_HUMAN	141 kDa	0.00	0.00	0.00	0.01	0.00	0.01	0	0	0	9	4	8	0	0	0	3	2	3	0	0	0	5.91%	2.83%	4.49%
201	26S protease regulatory subunit 7	PRS7_HUMAN	49 kDa	0.03	0.00	0.00	0.03	0.01	0.03	13	0	0	16	6	16	3	0	0	5	2	4	10.20%	0	0	15.70%	7.85%	11.80%
202	Annexin A2	ANXA2_HUMAN	39 kDa	0.05	0.00	0.00	0.05	0.02	0.02	18	0	0	23	8	6	4	0	0	6	2	2	15.30%	0	0	21.80%	7.67%	7.67%
203	Receptor-type tyrosine-protein phosphatase S	PTPRS_HUMAN	217 kDa	0.00	0.00	0.01	0.00	0.01	0.01	0	0	10	0	17	21	0	0	2	0	3	4	0	0	1.54%	0	2.72%	3.85%
204	Coatomer subunit beta'	COPB2_HUMAN	102 kDa	0.02	0.00	0.00	0.01	0.00	0.00	15	0	0	12	0	0	2	0	0	2	0	0	3.20%	0	0	3.20%	0	0
205	Protocadherin Fat 1	FAT1_HUMAN	506 kDa	0.00	0.00	0.00	0.00	0.00	0.00	0	0	0	5	0	5	0	0	0	2	0	2	0	0	0	0.55%	0	0.55%
206	Beta-actin-like protein 2	ACTBL_HUMAN	42 kDa	0.00	0.00	0.01	0.00	0.01	0.00	0	0	50	0	75	0	0	0	2	0	3	0	0	0	16.00%	0	18.10%	0
207	Nucleosome assembly protein 1-like 4	NP1L4_HUMAN	43 kDa	0.01	0.00	0.00	0.05	0.00	0.02	5	0	0	27	0	11	3	0	0	4	0	2	11.70%	0	0	16.00%	0	11.50%
208	Pleckstrin	PLEK_HUMAN	40 kDa	0.02	0.03	0.03	0.04	0.03	0.02	9	6	7	19	12	9	2	2	2	3	3	3	7.43%	7.43%	7.43%	11.70%	11.70%	11.70%
209	Coagulation factor X	FA10_HUMAN	55 kDa	0.00	0.00	0.00	0.00	0.02	0.00	0	0	0	0	10	0	0	0	0	0	2	0	0	0	0	0	4.10%	0
210	Cofilin-1	E9PK25_HUMAN	23 kDa	0.00	0.08	0.12	0.00	0.10	0.05	0	11	16	0	24	11	0	3	3	0	4	2	0	22.10%	22.10%	0	28.90%	22.10%
211	Hornerin	HORN_HUMAN	282 kDa	0.00	0.00	0.00	0.00	0.00	0.00	14	0	0	14	8	4	4	0	0	2	2	3	2.63%	0	0	1.23%	1.23%	1.72%
212	26S protease regulatory subunit 6B	PRS6B_HUMAN	47 kDa	0.04	0.00	0.00	0.04	0.01	0.04	18	0	0	22	5	20	3	0	0	3	2	3	20.10%	0	0	20.80%	12.40%	20.80%
213	Cathepsin B	CATB_HUMAN	38 kDa	0.08	0.00	0.00	0.01	0.00	0.00	29	0	0	6	0	0	4	0	0	3	0	0	15.30%	0	0	12.40%	0	0
214	Galactokinase	GALK1_HUMAN	42 kDa	0.00	0.00	0.03	0.00	0.02	0.03	0	0	7	0	8	12	0	0	2	0	2	2	0	0	6.12%	0	6.12%	6.12%
215	Tubulin beta-4A chain	TBB4A_HUMAN	50 kDa	0.02	0.00	0.02	0.03	0.03	0.03	102	0	81	155	144	130	3	0	2	3	2	3	51.60%	0	52.90%	60.40%	58.10%	56.50%
216	Elongation factor 1-delta	EF1D_HUMAN	31 kDa	0.04	0.00	0.00	0.04	0.01	0.00	11	0	0	14	4	0	2	0	0	2	2	0	8.54%	0	0	8.54%	8.54%	0
217	Complement component C8 beta chain	CO8B_HUMAN	67 kDa	0.03	0.00	0.00	0.01	0.00	0.00	17	0	0	9	0	0	3	0	0	2	0	0	5.58%	0	0	5.58%	0	0
218	Stress-induced-phosphoprotein 1	STIP1_HUMAN	63 kDa	0.00	0.00	0.00	0.01	0.00	0.00	0	0	0	4	0	0	0	0	0	2	0	0	0	0	0	4.24%	0	0
219	Purine nucleoside phosphorylase	PNPH_HUMAN	32 kDa	0.00	0.00	0.00	0.07	0.03	0.02	0	0	0	26	11	6	0	0	0	7	4	2	0	0	0	37.70%	13.10%	9.34%
220	ADP-ribosylation factor 1	ARF1_HUMAN (+1)	21 kDa	0.04	0.00	0.00	0.11	0.00	0.00	7	0	0	27	0	0	3	0	0	5	0	0	19.90%	0	0	37.60%	0	0
221	Proteasome activator complex subunit 2	PSME2_HUMAN	27 kDa	0.00	0.02	0.01	0.06	0.10	0.00	0	3	2	18	27	0	0	2	2	4	4	0	0	14.60%	14.60%	21.30%	21.30%	0
222	Cell division control protein 42 homolog	CDC42_HUMAN	21 kDa	0.00	0.00	0.00	0.00	0.05	0.00	0	0	0	0	10	0	0	0	0	0	2	0	0	0	0	0	15.70%	0
223	Protein disulfide-isomerase	PDIA1_HUMAN	57 kDa	0.01	0.00	0.00	0.03	0.02	0.01	8	0	0	18	11	7	2	0	0	7	2	2	8.27%	0	0	22.40%	8.27%	8.27%
224	Fructose-1,6-bisphosphatase 1	F16P1_HUMAN	37 kDa	0.00	0.00	0.00	0.00	0.04	0.04	0	0	0	0	16	15	0	0	0	0	2	2	0	0	0	0	8.28%	8.28%
225	Proteasome subunit alpha type-4	PSA4_HUMAN	29 kDa	0.07	0.00	0.00	0.03	0.00	0.00	21	0	0	11	0	0	6	0	0	3	0	0	16.50%	0	0	12.30%	0	0
226	Integrin beta-1	ITB1_HUMAN	88 kDa	0.00	0.00	0.00	0.01	0.00	0.01	0	0	0	3	1	5	0	0	0	2	2	4	0	0	0	1.13%	1.13%	4.64%
227	Capping protein (Actin filament) muscle Z-line, beta, isoform CRA_a	B1AK87_HUMAN (+1)	29 kDa	0.00	0.00	0.00	0.01	0.00	0.00	0	0	0	5	0	0	0	0	0	4	0	0	0	0	0	18.10%	0	0
228	Heterogeneous nuclear ribonucleoprotein H	G8JLB6_HUMAN	51 kDa	0.03	0.00	0.00	0.03	0.00	0.03	14	0	0	18	0	18	2	0	0	6	0	3	6.99%	0	0	12.70%	0	7.20%
229	Cathepsin L1	CATL1_HUMAN	38 kDa	0.00	0.00	0.00	0.02	0.00	0.00	0	0	0	9	0	0	0	0	0	3	0	0	0	0	0	10.80%	0	0
230	Tubulin beta-1 chain	TBB1_HUMAN	50 kDa	0.02	0.00	0.00	0.00	0.00	0.00	26	0	0	0	0	0	2	0	0	0	0	0	12.20%	0	0	0	0	0
231	HLA class I histocompatibility antigen, A-2 alpha chain	1A02_HUMAN	41 kDa	0.02	0.00	0.00	0.03	0.02	0.00	9	0	0	14	7	0	3	0	0	3	2	0	12.90%	0	0	15.10%	10.40%	0
232	Annexin A5	ANXA5_HUMAN	36 kDa	0.06	0.00	0.00	0.04	0.00	0.00	22	0	0	17	0	0	5	0	0	4	0	0	16.20%	0	0	11.60%	0	0
233	Hypoxia up-regulated protein 1	HYOU1_HUMAN	111 kDa	0.00	0.00	0.00	0.01	0.00	0.00	0	0	0	10	0	3	0	0	0	4	0	2	0	0	0	7.21%	0	3.90%
234	Beta-hexosaminidase subunit beta	HEXB_HUMAN	63 kDa	0.03	0.00	0.00	0.01	0.00	0.00	18	0	0	7	0	0	3	0	0	3	0	0	6.12%	0	0	6.12%	0	0
235	Cytosolic non-specific dipeptidase	CNDP2_HUMAN	53 kDa	0.00	0.02	0.03	0.01	0.02	0.02	0	5	8	4	12	9	0	2	2	2	4	2	0	5.89%	5.89%	5.89%	12.40%	5.89%
236	Alanine--tRNA ligase, cytoplasmic	SYAC_HUMAN	107 kDa	0.00	0.00	0.00	0.01	0.01	0.01	0	0	0	8	6	8	0	0	0	3	3	3	0	0	0	6.20%	6.71%	6.71%
237	Keratin, type II cytoskeletal 6A	K2C6A_HUMAN	60 kDa	0.03	0.00	0.00	0.00	0.00	0.01	90	0	0	0	0	47	6	0	0	0	0	2	23.90%	0	0	0	0	15.40%
238	Cytosolic 10-formyltetrahydrofolate dehydrogenase	AL1L1_HUMAN	99 kDa	0.00	0.00	0.00	0.00	0.00	0.01	0	0	0	0	0	7	0	0	0	0	0	3	0	0	0	0	0	3.22%
239	Septin-2	SEPT2_HUMAN	41 kDa	0.02	0.00	0.00	0.02	0.02	0.01	7	0	0	11	9	6	3	0	0	3	2	2	16.30%	0	0	12.50%	9.14%	9.14%
240	40S ribosomal protein S3	E9PL09_HUMAN (+1)	25 kDa	0.03	0.00	0.00	0.04	0.03	0.03	7	0	0	14	8	9	2	0	0	4	2	2	12.60%	0	0	20.80%	12.60%	12.60%
241	A disintegrin and metalloproteinase with thrombospondin motifs 13	ATS13_HUMAN	154 kDa	0.00	0.00	0.00	0.00	0.00	0.00	4	0	0	4	0	0	2	0	0	2	0	0	1.33%	0	0	1.33%	0	0
242	Keratin, type II cytoskeletal 1b	K2C1B_HUMAN	62 kDa	0.01	0.00	0.00	0.00	0.00	0.01	30	0	0	0	0	21	3	0	0	0	0	2	12.60%	0	0	0	0	6.23%

243	26S protease regulatory subunit 6A	PRS6A_HUMAN	49 kDa	0.01	0.00	0.00	0.02	0.00	0.02	6	0	0	11	0	9	2	0	0	3	0	3	7.06%	0	0	14.60%	0	12.10%
244	Septin-7	E7EPK1_HUMAN (+2)	51 kDa	0.03	0.00	0.00	0.01	0.00	0.01	16	0	0	8	0	5	2	0	0	3	0	2	7.55%	0	0	11.40%	0	4.35%
245	Coronin-1C	COR1C_HUMAN	53 kDa	0.00	0.00	0.00	0.02	0.03	0.02	0	0	0	14	14	11	0	0	0	4	2	2	0	0	0	14.30%	8.23%	8.23%
246	Peroxiredoxin-1 (Fragment)	A0A0A0MSI0_HUMAN	19 kDa	0.00	0.00	0.03	0.02	0.08	0.00	0	0	3	5	15	0	0	0	2	2	3	0	0	0	11.70%	11.70%	16.40%	0
247	UDP-glucose 6-dehydrogenase	UGDH_HUMAN	55 kDa	0.00	0.00	0.00	0.00	0.01	0.02	0	0	0	3	8	11	0	0	0	2	2	3	0	0	0	6.88%	6.88%	9.51%
248	EMILIN-2	EMIL2_HUMAN	116 kDa	0.01	0.00	0.00	0.00	0.00	0.00	13	0	0	0	0	0	4	0	0	0	0	0	8.45%	0	0	0	0	0
249	Zyxin (Fragment)	H0Y2Y8_HUMAN (+1)	58 kDa	0.00	0.00	0.00	0.01	0.01	0.00	0	0	0	4	6	3	0	0	0	2	3	2	0	0	0	7.59%	11.10%	8.70%
250	WD repeat-containing protein 1	WDR1_HUMAN	66 kDa	0.00	0.00	0.00	0.01	0.00	0.00	0	0	0	9	0	0	0	0	0	2	0	0	0	0	0	3.80%	0	0
251	Putative Ras-related protein Rab-1C	RAB1C_HUMAN	22 kDa	0.00	0.00	0.00	0.04	0.00	0.00	0	0	0	8	0	0	0	0	0	4	0	0	0	0	0	13.40%	0	0
252	Vasodilator-stimulated phosphoprotein	VASP_HUMAN	40 kDa	0.00	0.00	0.00	0.02	0.00	0.00	0	0	0	9	0	0	0	0	0	2	0	0	0	0	0	7.63%	0	0
253	26S proteasome non-ATPase regulatory subunit 3	PSMD3_HUMAN	61 kDa	0.02	0.00	0.00	0.02	0.00	0.00	10	0	0	11	0	0	2	0	0	2	0	0	4.87%	0	0	4.87%	0	0
254	Proteasome subunit alpha type-5	PSA5_HUMAN	26 kDa	0.08	0.00	0.00	0.04	0.00	0.00	21	0	0	13	0	0	4	0	0	4	0	0	24.10%	0	0	24.10%	0	0
255	Galectin-3-binding protein	LG3BP_HUMAN	65 kDa	0.03	0.00	0.00	0.00	0.00	0.00	22	0	0	0	0	0	6	0	0	0	0	0	12.30%	0	0	0	0	0
256	26S proteasome non-ATPase regulatory subunit 11	PSD11_HUMAN	47 kDa	0.02	0.00	0.00	0.01	0.00	0.02	11	0	0	7	0	8	3	0	0	3	0	2	8.53%	0	0	8.06%	0	5.92%
257	Tyrosine--tRNA ligase, cytoplasmic	SYYC_HUMAN	59 kDa	0.00	0.00	0.00	0.01	0.02	0.02	0	0	0	5	9	11	0	0	0	2	2	2	0	0	0	6.63%	6.25%	6.25%
258	Calreticulin	CALR_HUMAN	48 kDa	0.00	0.00	0.00	0.02	0.01	0.02	0	0	0	9	7	10	0	0	0	2	2	2	0	0	0	15.60%	15.60%	15.60%
259	Coatomer subunit beta	COPB_HUMAN	107 kDa	0.00	0.00	0.00	0.00	0.00	0.00	0	0	0	2	0	0	0	0	0	2	0	0	0	0	0	3.78%	0	0
260	Spliceosome RNA helicase DDX39B	DX39B_HUMAN	49 kDa	0.00	0.00	0.00	0.03	0.03	0.02	0	0	0	20	14	9	0	0	0	3	3	3	0	0	0	8.41%	8.41%	8.41%
261	Proteasome subunit beta type-6	PSB6_HUMAN	25 kDa	0.02	0.00	0.00	0.01	0.00	0.00	5	0	0	4	0	0	2	0	0	2	0	0	8.79%	0	0	8.79%	0	0
262	Thyroxine-binding globulin	THBG_HUMAN	46 kDa	0.01	0.03	0.00	0.00	0.00	0.00	5	9	0	0	0	0	2	2	0	0	0	0	4.82%	4.82%	0	0	0	0
263	Protein C1QTNF3-AMACR	E9PGA6_HUMAN	31 kDa	0.00	0.05	0.00	0.00	0.00	0.00	0	9	0	0	0	0	0	2	0	0	0	0	0	8.45%	0	0	0	0
264	Collagen alpha-1(V) chain	CO5A1_HUMAN	184 kDa	0.00	0.00	0.01	0.00	0.00	0.00	0	3	7	0	0	0	0	2	2	0	0	0	0	2.12%	2.23%	0	0	0
265	Ig heavy chain V-III region HIL	HV310_HUMAN	14 kDa	0.00	0.00	0.00	0.02	0.00	0.00	0	0	0	4	0	0	0	0	0	3	0	0	0	0	0	12.40%	0	0
266	Cathepsin Z	CATZ_HUMAN	34 kDa	0.00	0.00	0.00	0.00	0.03	0.00	0	0	0	0	12	0	0	0	0	0	3	0	0	0	0	0	8.91%	0
267	Neutral alpha-glucosidase AB	GANAB_HUMAN	107 kDa	0.00	0.00	0.00	0.01	0.00	0.00	5	0	0	18	0	5	4	0	0	6	0	3	6.78%	0	0	9.53%	0	5.40%
268	26S protease regulatory subunit 8	PRS8_HUMAN	46 kDa	0.03	0.00	0.00	0.02	0.00	0.02	13	0	0	12	0	7	3	0	0	3	0	3	12.30%	0	0	12.60%	0	12.60%
269	Lupus La protein	LA_HUMAN	47 kDa	0.00	0.00	0.00	0.03	0.02	0.02	0	0	0	14	9	11	0	0	0	3	4	3	0	0	0	5.88%	12.50%	9.07%
270	Haptoglobin	A0A087WU08_HUMAI	31 kDa	0.04	0.00	0.00	0.03	0.00	0.00	12	0	0	10	0	0	2	0	0	2	0	0	6.05%	0	0	6.05%	0	0
271	Keratin, type II cytoskeletal 4	K2C4_HUMAN	57 kDa	0.00	0.07	0.00	0.00	0.00	0.00	0	26	0	0	0	0	0	7	0	0	0	0	0	17.00%	0	0	0	0
272	Myosin light polypeptide 6	G8JLA2_HUMAN (+1)	17 kDa	0.01	0.00	0.00	0.01	0.00	0.00	2	0	0	2	0	0	2	0	0	2	0	0	19.10%	0	0	19.10%	0	0
273	Cytoplasmic aconitate hydratase	ACOC_HUMAN	98 kDa	0.00	0.00	0.00	0.00	0.00	0.01	0	0	0	0	0	14	0	0	0	0	0	3	0	0	0	0	0	3.71%
274	26S proteasome non-ATPase regulatory subunit 14	PSDE_HUMAN	35 kDa	0.03	0.00	0.00	0.02	0.01	0.00	9	0	0	7	5	0	2	0	0	3	2	0	19.40%	0	0	19.40%	19.40%	0
275	General vesicular transport factor p115	USO1_HUMAN	108 kDa	0.00	0.00	0.00	0.01	0.00	0.00	0	0	0	9	0	0	0	0	0	2	0	0	0	0	0	3.12%	0	0
276	Argininosuccinate lyase	ARLY_HUMAN	52 kDa	0.00	0.00	0.00	0.00	0.00	0.01	0	0	0	0	0	5	0	0	0	0	0	2	0	0	0	0	0	4.96%
277	Myosin-4	MYH4_HUMAN	223 kDa	0.00	0.00	0.00	0.00	0.00	0.00	0	0	0	0	0	1	0	0	0	0	0	2	0	0	0	0	0	0.62%
278	Non-POU domain-containing octamer-binding protein	NONO_HUMAN	54 kDa	0.00	0.00	0.00	0.00	0.00	0.01	0	0	0	0	0	6	0	0	0	0	0	3	0	0	0	0	0	11.50%
279	Kynureninase	KYNU_HUMAN	52 kDa	0.00	0.00	0.00	0.03	0.04	0.00	0	0	0	16	20	0	0	0	0	4	4	0	0	0	0	12.70%	12.70%	0
280	Proteasome subunit beta type-10	PSB10_HUMAN	29 kDa	0.08	0.00	0.00	0.00	0.00	0.00	24	0	0	0	0	0	3	0	0	0	0	0	19.40%	0	0	0	0	0
281	Amyloid-like protein 2	APLP2_HUMAN	87 kDa	0.01	0.01	0.00	0.00	0.00	0.00	11	3	0	0	0	0	3	2	0	0	0	0	4.19%	4.19%	0	0	0	0
282	Neuropilin-1	E9PEP6_HUMAN (+1)	101 kDa	0.00	0.00	0.00	0.00	0.00	0.01	0	0	0	0	0	7	0	0	0	0	0	2	0	0	0	0	0	4.08%
283	14-3-3 protein beta/alpha	1433B_HUMAN	28 kDa	0.00	0.00	0.06	0.00	0.05	0.00	0	0	21	0	32	0	0	0	3	0	3	0	0	0	17.50%	0	21.10%	0
284	Actin-related protein 2/3 complex subunit 3	ARPC3_HUMAN	21 kDa	0.06	0.00	0.00	0.00	0.00	0.00	12	0	0	0	0	0	2	0	0	0	0	0	13.50%	0	0	0	0	0
285	Integrin-linked protein kinase	A0A0A0MTH3_HUMAI	55 kDa	0.01	0.00	0.00	0.01	0.00	0.00	3	0	0	7	0	0	3	0	0	4	0	0	6.63%	0	0	9.52%	0	0
286	D-3-phosphoglycerate dehydrogenase	SERA_HUMAN	57 kDa	0.00	0.00	0.00	0.01	0.00	0.00	0	0	0	7	0	0	0	0	0	2	0	0	0	0	0	4.32%	0	0
287	26S protease regulatory subunit 4	PRS4_HUMAN	49 kDa	0.00	0.00	0.00	0.00	0.00	0.01	0	0	0	0	0	6	0	0	0	0	0	3	0	0	0	0	0	11.10%
288	26S protease regulatory subunit 10B	A0A087X2I1_HUMAN	46 kDa	0.02	0.00	0.00	0.02	0.00	0.00	7	0	0	10	0	0	2	0	0	2	0	0	7.20%	0	0	7.20%	0	0
289	Interferon-induced GTP-binding protein Mx2	MX2_HUMAN	82 kDa	0.00	0.00	0.00	0.02	0.00	0.00	0	0	0	21	0	0	0	0	0	3	0	0	0	0	0	5.17%	0	0
290	Importin-7	IPO7_HUMAN	120 kDa	0.00	0.00	0.00	0.00	0.00	0.01	0	0	0	0	0	9	0	0	0	0	0	2	0	0	0	0	0	2.50%
291	14-3-3 protein theta	1433T_HUMAN	28 kDa	0.00	0.04	0.03	0.01	0.03	0.00	0	15	16	15	20	0	0	2	2	2	2	0	0	17.60%	17.60%	17.60%	17.60%	0
292	Exportin-2	XPO2_HUMAN	110 kDa	0.00	0.00	0.00	0.01	0.00	0.00	0	0	0	13	0	0	0	0	0	2	0	0	0	0	0	2.68%	0	0
293	Kinectin	KTN1_HUMAN	156 kDa	0.00	0.00	0.00	0.00	0.00	0.00	0	0	0	5	0	0	0	0	0	3	0	0	0	0	0	3.68%	0	0

294	Proteasome subunit beta type-2	PSB2_HUMAN	23 kDa	0.05	0.00	0.00	0.00	0.00	0.00	10	0	0	0	0	0	3	0	0	0	0	0	13.40%	0	0	0	0	0
295	Glucose-6-phosphate 1-dehydrogenase	G6PD_HUMAN	59 kDa	0.00	0.00	0.00	0.01	0.00	0.00	0	0	0	8	0	0	0	0	0	3	0	0	0	0	0	8.16%	0	0
296	Keratin, type I cytoskeletal 13	K1C13_HUMAN	50 kDa	0.00	0.07	0.00	0.00	0.00	0.00	0	29	0	0	0	0	0	6	0	0	0	0	0	20.70%	0	0	0	0
297	Actin-related protein 2/3 complex subunit 5	ARPC5_HUMAN	16 kDa	0.09	0.00	0.00	0.03	0.00	0.00	14	0	0	6	0	0	3	0	0	2	0	0	23.80%	0	0	15.90%	0	0
298	Homogentisate 1,2-dioxygenase	HGD_HUMAN	50 kDa	0.00	0.00	0.02	0.00	0.00	0.01	0	0	6	0	0	3	0	0	2	0	0	2	0	0	10.10%	0	0	10.10%
299	Nuclease-sensitive element-binding protein 1	YBOX1_HUMAN	36 kDa	0.00	0.00	0.00	0.02	0.00	0.00	0	0	0	8	0	0	0	0	0	2	0	0	0	0	0	13.00%	0	0
300	EH domain-containing protein 3	A0A0C4DH83_HUMAN	62 kDa	0.02	0.00	0.00	0.00	0.00	0.00	11	0	0	0	0	0	2	0	0	0	0	0	6.04%	0	0	0	0	0
301	60S acidic ribosomal protein P2	RLA2_HUMAN	12 kDa	0.00	0.00	0.00	0.00	0.03	0.00	0	0	0	0	4	0	0	0	0	0	2	0	0	0	0	0	39.10%	0
302	26S proteasome non-ATPase regulatory subunit 12	PSD12_HUMAN	53 kDa	0.02	0.00	0.00	0.00	0.00	0.00	9	0	0	0	0	0	2	0	0	0	0	0	7.89%	0	0	0	0	0
303	Vacuolar protein sorting-associated protein 35	VPS35_HUMAN	92 kDa	0.00	0.00	0.00	0.00	0.00	0.00	3	0	0	4	0	0	2	0	0	2	0	0	3.14%	0	0	3.14%	0	0
304	Ig heavy chain V-III region WEA	HV302_HUMAN	12 kDa	0.00	0.00	0.03	0.00	0.00	0.00	0	0	2	0	0	0	0	0	2	0	0	0	0	0	19.30%	0	0	0
305	Programmed cell death 6-interacting protein	PDC6I_HUMAN	96 kDa	0.00	0.00	0.00	0.00	0.01	0.01	0	0	0	4	6	6	0	0	0	2	2	2	0	0	0	2.76%	3.34%	3.34%
306	Coatomer subunit alpha	COPA_HUMAN	138 kDa	0.00	0.00	0.00	0.00	0.00	0.00	3	0	0	0	0	0	2	0	0	0	0	0	2.21%	0	0	0	0	0
307	Heat shock protein 105 kDa	HS105_HUMAN	97 kDa	0.00	0.00	0.00	0.00	0.00	0.00	0	0	0	4	0	0	0	0	0	2	0	0	0	0	0	3.61%	0	0
308	Calcium/calmodulin-dependent protein kinase (CaM kinase) II delta, isoform CRA_e	D6R938_HUMAN (+3)	56 kDa	0.01	0.00	0.00	0.00	0.00	0.00	8	0	0	0	0	0	4	0	0	0	0	0	12.00%	0	0	0	0	0
309	Adenosine kinase	ADK_HUMAN	41 kDa	0.00	0.00	0.01	0.00	0.00	0.01	0	0	3	0	0	5	0	0	2	0	0	2	0	0	4.97%	0	0	5.52%
310	Proteasome activator complex subunit 1	PSME1_HUMAN	29 kDa	0.00	0.00	0.00	0.02	0.03	0.00	0	0	0	7	9	0	0	0	0	2	2	0	0	0	0	10.40%	10.40%	0
311	Phosphoglucomutase-1	PGM1_HUMAN	61 kDa	0.00	0.00	0.00	0.00	0.00	0.00	0	0	0	0	0	3	0	0	0	0	0	2	0	0	0	0	0	4.63%
312	V-type proton ATPase subunit B, brain isoform	VATB2_HUMAN	57 kDa	0.00	0.00	0.00	0.01	0.00	0.00	0	0	0	9	0	0	0	0	0	4	0	0	0	0	0	9.39%	0	0
313	Tropomyosin alpha-4 chain	TPM4_HUMAN	29 kDa	0.00	0.00	0.00	0.00	0.04	0.00	0	0	0	0	22	0	0	0	0	0	2	0	0	0	0	0	17.70%	0
314	Splicing factor, proline- and glutamine-rich	SFPQ_HUMAN	76 kDa	0.00	0.00	0.00	0.00	0.01	0.00	0	0	0	0	6	0	0	0	0	0	2	0	0	0	0	0	4.95%	0
315	Protein kinase C and casein kinase substrate in neurons protein 2	PACN2_HUMAN	56 kDa	0.00	0.00	0.00	0.01	0.00	0.00	0	0	0	6	0	0	0	0	0	3	0	0	0	0	0	5.97%	0	0
316	Alpha-amylase 1	AMY1_HUMAN	58 kDa	0.00	0.00	0.01	0.00	0.00	0.00	0	0	5	0	0	0	0	0	2	0	0	0	0	0	4.89%	0	0	0
317	Integrin beta-2	ITB2_HUMAN	85 kDa	0.01	0.00	0.00	0.00	0.00	0.00	4	0	0	0	0	0	3	0	0	0	0	0	3.12%	0	0	0	0	0
318	Tripeptidyl-peptidase 1	A0A0C4DGZ9_HUMAN	60 kDa	0.00	0.00	0.00	0.02	0.00	0.00	0	0	0	12	0	0	0	0	0	4	0	0	0	0	0	10.80%	0	0
319	KH domain-containing, RNA-binding, signal transduction-associated protein 1	KHDR1_HUMAN	48 kDa	0.00	0.00	0.00	0.00	0.02	0.01	0	0	0	0	9	7	0	0	0	0	2	2	0	0	0	0	7.67%	7.67%
320	Hydroxyacyl-coenzyme A dehydrogenase, mitochondrial	E9PF18_HUMAN	35 kDa	0.01	0.00	0.00	0.00	0.00	0.00	4	0	0	0	0	0	2	0	0	0	0	0	16.40%	0	0	0	0	0
321	Pyridoxal kinase	F2Z2Y4_HUMAN (+1)	31 kDa	0.00	0.00	0.00	0.03	0.00	0.00	0	0	0	12	0	0	0	0	0	3	0	0	0	0	0	14.00%	0	0
322	Ribosome-binding protein 1	A0A0A0MRV0_HUMAI	152 kDa	0.00	0.00	0.00	0.00	0.00	0.00	0	0	0	2	0	0	0	0	0	2	0	0	0	0	0	2.20%	0	0
323	Dipeptidyl peptidase 2	DPP2_HUMAN	54 kDa	0.00	0.00	0.00	0.01	0.00	0.00	0	0	0	5	0	0	0	0	0	2	0	0	0	0	0	5.89%	0	0
324	Guanine nucleotide-binding protein subunit beta-2-like 1	GBLP_HUMAN	35 kDa	0.00	0.00	0.00	0.00	0.00	0.00	0	0	0	2	0	0	0	0	0	2	0	0	0	0	0	8.83%	0	0
325	CTP synthase 1	PYRG1_HUMAN	67 kDa	0.00	0.00	0.00	0.01	0.00	0.00	0	0	0	6	0	0	0	0	0	4	0	0	0	0	0	8.80%	0	0
326	Eukaryotic translation initiation factor 3 subunit I	EIF3I_HUMAN	37 kDa	0.01	0.00	0.00	0.01	0.00	0.00	3	0	0	5	0	0	2	0	0	3	0	0	8.62%	0	0	13.20%	0	0
327	C-1-tetrahydrofolate synthase, cytoplasmic	F5H2F4_HUMAN	111 kDa	0.00	0.00	0.00	0.00	0.00	0.00	0	0	0	0	0	3	0	0	0	0	0	2	0	0	0	0	0	2.35%
328	Hepatocyte growth factor activator	HGFA_HUMAN	71 kDa	0.00	0.00	0.00	0.00	0.00	0.01	0	0	0	0	0	8	0	0	0	0	0	2	0	0	0	0	0	3.51%
329	Macrophage-capping protein	CAPG_HUMAN	38 kDa	0.00	0.00	0.00	0.00	0.02	0.00	0	0	0	0	9	0	0	0	0	0	2	0	0	0	0	0	10.90%	0
330	Syntenin-1	SDCB1_HUMAN	32 kDa	0.02	0.00	0.00	0.01	0.00	0.00	7	0	0	5	0	0	4	0	0	3	0	0	23.80%	0	0	23.80%	0	0
331	Asparagine--tRNA ligase, cytoplasmic	SYNC_HUMAN	63 kDa	0.00	0.00	0.00	0.01	0.00	0.00	0	0	0	5	0	3	0	0	0	3	0	2	0	0	0	8.39%	0	5.66%
332	Zinc-alpha-2-glycoprotein	ZA2G_HUMAN	34 kDa	0.03	0.00	0.00	0.00	0.00	0.00	9	0	0	0	0	0	2	0	0	0	0	0	12.40%	0	0	0	0	0
333	Triosephosphate isomerase	TPIS_HUMAN	31 kDa	0.00	0.00	0.03	0.00	0.00	0.00	0	0	6	0	0	0	0	0	2	0	0	0	0	0	9.79%	0	0	0
334	Eukaryotic translation initiation factor 2 subunit 1	IF2A_HUMAN	36 kDa	0.01	0.00	0.00	0.00	0.01	0.00	3	0	0	0	3	0	2	0	0	0	2	0	9.21%	0	0	0	9.21%	0
335	Nicotinate phosphoribosyltransferase	G5E977_HUMAN	62 kDa	0.00	0.00	0.00	0.00	0.00	0.00	0	0	0	3	0	0	0	0	0	2	0	0	0	0	0	6.34%	0	0

336	Signal transducer and activator of transcription 1-alpha/beta	STAT1_HUMAN	87 kDa	0.00	0.00	0.00	0.00	0.00	0.00	0	0	0	3	0	0	0	0	0	2	0	0	0	0	0	3.33%	0	0
337	Guanine nucleotide-binding protein G(I)/G(S)/G(T) subunit beta-2	E7EP32_HUMAN (+1)	32 kDa	0.00	0.00	0.00	0.01	0.00	0.00	0	0	0	3	0	0	0	0	0	2	0	0	0	0	0	6.76%	0	0
338	Glycogen phosphorylase, brain form	PYGB_HUMAN	97 kDa	0.00	0.00	0.00	0.00	0.00	0.00	0	0	0	5	0	0	0	0	0	2	0	0	0	0	0	3.44%	0	0
339	Malate dehydrogenase, mitochondrial	MDHM_HUMAN	36 kDa	0.00	0.00	0.00	0.01	0.00	0.00	0	0	0	4	0	0	0	0	0	3	0	0	0	0	0	11.20%	0	0
340	Serine--tRNA ligase, cytoplasmic	SYSC_HUMAN	59 kDa	0.00	0.00	0.00	0.00	0.00	0.00	0	0	0	3	0	0	0	0	0	2	0	0	0	0	0	7.98%	0	0
341	Procollagen-lysine,2-oxoglutarate 5-dioxygenase 1	PLOD1_HUMAN	84 kDa	0.00	0.00	0.00	0.00	0.00	0.00	2	0	0	0	0	0	2	0	0	0	0	0	4.26%	0	0	0	0	0
342	V-type proton ATPase subunit E 1	VATE1_HUMAN	26 kDa	0.00	0.00	0.00	0.01	0.00	0.00	0	0	0	4	0	0	0	0	0	3	0	0	0	0	0	15.00%	0	0
343	Serglycin	SRGN_HUMAN	18 kDa	0.02	0.00	0.00	0.00	0.00	0.00	4	0	0	0	0	0	2	0	0	0	0	0	10.10%	0	0	0	0	0
344	Neural cell adhesion molecule 1	NCAM1_HUMAN	95 kDa	0.00	0.00	0.00	0.00	0.00	0.00	0	0	0	0	2	0	0	0	0	0	2	0	0	0	0	0	2.91%	0
345	Proteasome subunit beta type-7	PSB7_HUMAN	30 kDa	0.01	0.00	0.00	0.00	0.00	0.00	3	0	0	0	0	0	2	0	0	0	0	0	7.58%	0	0	0	0	0
346	ADAM DEC1	ADEC1_HUMAN	53 kDa	0.00	0.00	0.00	0.00	0.02	0.00	0	0	0	0	8	0	0	0	0	0	2	0	0	0	0	0	8.72%	0
347	Protein SET	SET_HUMAN	33 kDa	0.00	0.00	0.00	0.00	0.02	0.00	0	0	0	0	5	0	0	0	0	0	3	0	0	0	0	0	12.80%	0
348	Obg-like ATPase 1	J3KQ32_HUMAN (+1)	47 kDa	0.00	0.00	0.00	0.01	0.00	0.00	0	0	0	8	0	0	0	0	0	2	0	0	0	0	0	6.73%	0	0
349	Desmocollin-1	DSC1_HUMAN	100 kDa	0.00	0.00	0.00	0.00	0.00	0.00	2	0	0	0	0	0	2	0	0	0	0	0	3.13%	0	0	0	0	0
350	Fascin	FSCN1_HUMAN	55 kDa	0.00	0.00	0.00	0.01	0.00	0.00	0	0	0	4	0	0	0	0	0	2	0	0	0	0	0	6.90%	0	0
351	Prolactin-inducible protein	PIP_HUMAN	17 kDa	0.03	0.00	0.00	0.00	0.00	0.00	4	0	0	0	0	0	2	0	0	0	0	0	17.80%	0	0	0	0	0
352	Ferritin light chain	FRIL_HUMAN	20 kDa	0.00	0.00	0.00	0.00	0.02	0.00	0	0	0	0	3	0	0	0	0	0	2	0	0	0	0	0	17.70%	0
353	Reticulocalbin-3 (Fragment)	M0QZH0_HUMAN (+1)	20 kDa	0.00	0.00	0.02	0.00	0.00	0.00	0	0	2	0	0	0	0	0	2	0	0	0	0	0	14.80%	0	0	0
354	Keratin, type II cytoskeletal 6B	K2C6B_HUMAN	60 kDa	0.00	0.00	0.00	0.00	0.00	0.00	114	0	0	0	0	0	2	0	0	0	0	0	25.70%	0	0	0	0	0
355	Superoxide dismutase	A0A0C4DFU2_HUMAN	25 kDa	0.00	0.00	0.00	0.00	0.01	0.00	0	0	0	0	3	0	0	0	0	0	2	0	0	0	0	0	12.60%	0

END OF FILE

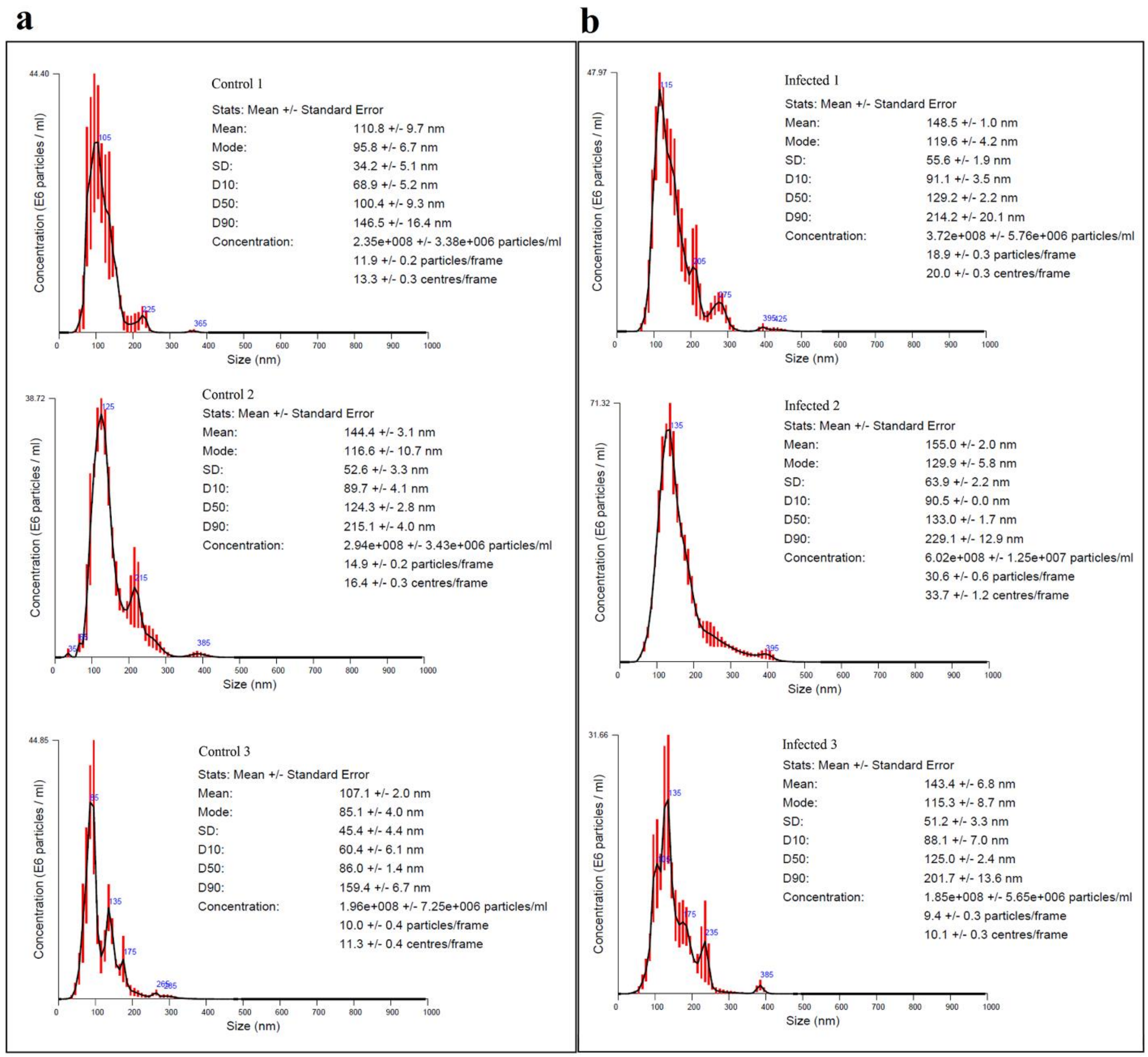

Supplementary Figure 1. Nanoparticle analysis of exosome samples. Representative histograms of each biological replicate of exosome derived from: a. *Mtb*-infected cells and b. Control cells.

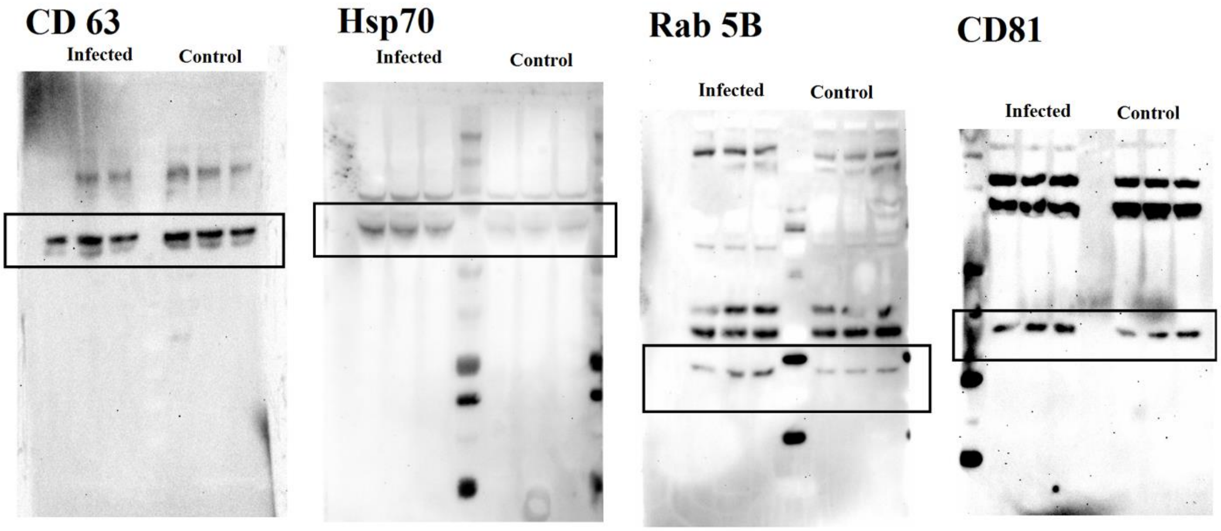

Supplementary Figure 2. Original images of the western blot used in Fig. 1d. Western blot detected using the chemiluminescent substrate Super signal West Pico.

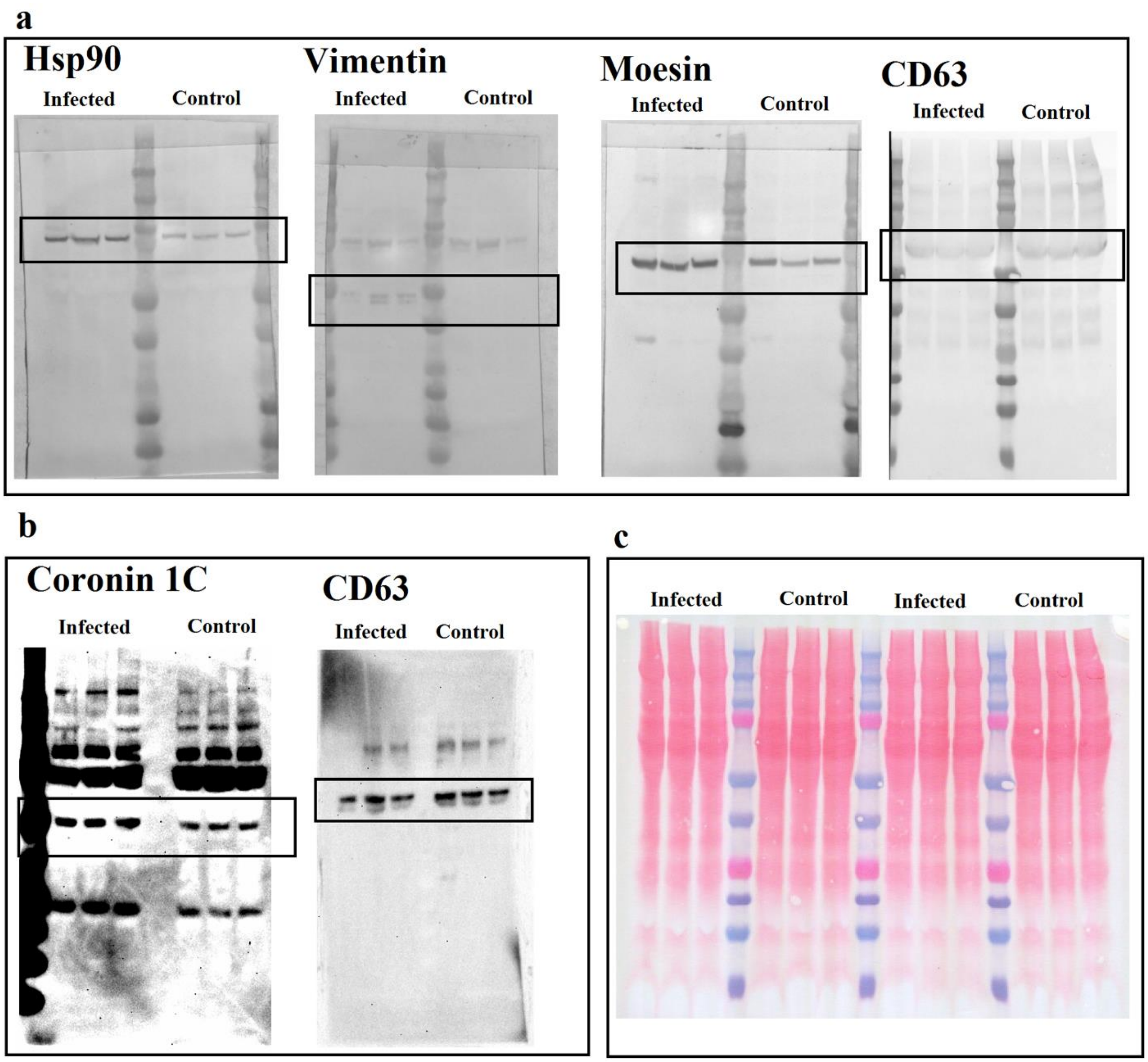

Supplementary Figure 3. Original images of the western blot reported in Figure 3. a) Western blot detected using the chromogenic substrate 4-chloro 1-naphtol. b) Western blot detected using the chemiluminescent substrate Super signal West Pico. c) Membrane for the western blot to detect Hsp70 (left side: infected and control) and CD63 (right side infected and control) the membrane was stained with Ponceau S to show equal protein loading of 50 ug.
